# Effects of Titanium Dioxide Nanoparticles on Porcine Prepubertal Sertoli Cells: An “*In Vitro*” Study

**DOI:** 10.3389/fendo.2021.751915

**Published:** 2022-01-03

**Authors:** Francesca Mancuso, Iva Arato, Alessandro Di Michele, Cinzia Antognelli, Luca Angelini, Catia Bellucci, Cinzia Lilli, Simona Boncompagni, Aurora Fusella, Desirée Bartolini, Carla Russo, Massimo Moretti, Morena Nocchetti, Angela Gambelunghe, Giacomo Muzi, Tiziano Baroni, Stefano Giovagnoli, Giovanni Luca

**Affiliations:** ^1^ Department of Medicine and Surgery, University of Perugia, Perugia, Italy; ^2^ Department of Physics and Geology, University of Perugia, Perugia, Italy; ^3^ International Biotechnological Center for Endocrine, Metabolic and Embryo-Reproductive Translational Research (CIRTEMER), Department of Medicine and Surgery, University of Perugia, Perugia, Italy; ^4^ Center for Advanced Studies and Technology (CAST) and Department of Neuroscience, Imaging and Clinical Sciences (DNICS), University G. d’Annunzio (Ud’A) of Chieti-Pescara, Chieti, Italy; ^5^ Department of Pharmaceutical Sciences, University of Perugia, Perugia, Italy; ^6^ Division of Medical Andrology and Endocrinology of Reproduction, Saint Mary Hospital, Terni, Italy

**Keywords:** ROS, comet, antioxidant enzymes, proinflammatory pathways, Sertoli cells, titanium dioxide nanoparticles

## Abstract

The increasing use of nanomaterials in a variety of industrial, commercial, medical products, and their environmental spreading has raised concerns regarding their potential toxicity on human health. Titanium dioxide nanoparticles (TiO_2_ NPs) represent one of the most commonly used nanoparticles. Emerging evidence suggested that exposure to TiO_2_ NPs induced reproductive toxicity in male animals. In this *in vitro* study, porcine prepubertal Sertoli cells (SCs) have undergone acute (24 h) and chronic (from 1 up to 3 weeks) exposures at both subtoxic (5 µg/ml) and toxic (100 µg/ml) doses of TiO_2_ NPs. After performing synthesis and characterization of nanoparticles, we focused on SCs morphological/ultrastructural analysis, apoptosis, and functionality (AMH, inhibin B), ROS production and oxidative DNA damage, gene expression of antioxidant enzymes, proinflammatory/immunomodulatory cytokines, and MAPK kinase signaling pathway. We found that 5 µg/ml TiO_2_ NPs did not induce substantial morphological changes overtime, but ultrastructural alterations appeared at the third week. Conversely, SCs exposed to 100 µg/ml TiO_2_ NPs throughout the whole experiment showed morphological and ultrastructural modifications. TiO_2_ NPs exposure, at each concentration, induced the activation of caspase-3 at the first and second week. AMH and inhibin B gene expression significantly decreased up to the third week at both concentrations of nanoparticles. The toxic dose of TiO_2_ NPs induced a marked increase of intracellular ROS and DNA damage at all exposure times. At both concentrations, the increased gene expression of antioxidant enzymes such as SOD and HO-1 was observed whereas, at the toxic dose, a clear proinflammatory stress was evaluated along with the steady increase in the gene expression of IL-1α and IL-6. At both concentrations, an increased phosphorylation ratio of p-ERK1/2 was observed up to the second week followed by the increased phosphorylation ratio of p-NF-kB in the chronic exposure. Although *in vitro*, this pilot study highlights the adverse effects even of subtoxic dose of TiO_2_ NPs on porcine prepubertal SCs functionality and viability and, more importantly, set the basis for further *in vivo* studies, especially in chronic exposure at subtoxic dose of TiO_2_ NPs, a condition closer to the human exposure to this nanoagent.

## 1 Introduction

The use of nanoparticles (NPs) has steadily increased over the past decade, due to their unique physical and chemical properties (1, 2) and has raised growing concern about their possible effects on human health (3). Given the small size and biocompatibility, NPs are potentially able to enter the body through different routes, such as inhalation, ingestion, skin uptake, injection, or implantation and interfere with several cellular physiological processes (4, 5). In particular, chronic exposure to NPs is associated with different disorders in animals, including pulmonary injury, hepatotoxicity, neurotoxicity, renal toxicity, and irreversible testis damage (6–12). TiO_2_ NPs are among the top 5 NPs used in consumer products. In fact, they are added to cosmetics, chewing gum, beverage, sauces, printing ink, paper, sunscreens, car materials, and decomposing organic matters in water purification (13–15). It has been shown that TiO_2_ NPs can be accumulated in the kidney (0.418 µg/g tissue), liver (5.78 μg/g tissue), lung (4.02 µg/g tissue), spleen (19.16 µg/g tissue), brain (145 ng/g tissue), and reproductive organs in animal models (75 ng/g tissue) (16–19).

A number of *in vivo* studies in mice or rats demonstrated that TiO_2_ NPs are able to cross blood–testis barrier and accumulate in the testis resulting in the reduction of sperm numbers and motility, increased sperm morphological abnormalities, and germ cell apoptosis with histopathological changes in the testis and marked decrease in serum testosterone, LH, and FSH levels (20–24). Komatsu et al. (25) demonstrated that TiO_2_ NPs affected the viability, proliferation, and gene expressions of mouse Leydig TM3 cells, the testosterone-producing cells of the testis. However, the molecular mechanisms of the observed impaired spermatogenesis nowadays are still discussed (26).

SCs play a critical role in the testis, providing the structural and metabolic supports for nourishing and developing germ cells by secreting many essential factors. Hence, any chemical agent that decreases the viability and the function of SCs may produce adverse effects on spermatogenesis and male infertility.

The very few studies describing the detrimental effect of TiO_2_ NPs on SCs have been performed in mice and rat models under acute exposures (26–28). However, these experimental conditions may greatly differ from those under which human exposure can potentially occur and often result in conflicting data that are not applicable for risk assessment studies (29). Hence, to realistically mimic long-term exposure for toxicity testing, we here assessed the effect of TiO_2_ NPs either under a subtoxic (5 µg/ml) or toxic dose (100 µg/ml) in an acute (24 h) and chronic exposure (up to 3 weeks), on an *in vitro* model of porcine prepubertal SCs, an experimental animal model sharing significant physiological similarity with humans. The concentrations were chosen according to 3-(4,5-dimethylthiazol-2-yl)-2,5-diphenyl-2H-tetrazolium bromide (MTT) results in our preliminary cytotoxicity tests as reported in the **Supplementary Material** and on the basis of literature data (26, 27).

Light microscopy and transmission electron microscopy (TEM) analysis revealed that TiO_2_ NPs caused significant morphological and ultrastructural alterations at the toxic dose but, at subtoxic dose, ultrastructural alterations appeared at the third week.

The evaluation of caspase-3, as crucial mediator of apoptosis, showed at each concentration of NPs, an increase at the first and second week, with the cleavage of p35 into the p19 kDa active fragment.

TiO_2_ NPs exposure negatively affected SCs functionality at both concentrations of NPs and induced reactive oxygen species (ROS) production and DNA oxidative damage at the toxic dose.

At both concentrations, an increased gene expression of antioxidant enzymes such as superoxide dismutase (SOD), heme oxygenase (HO-1) was observed, whereas, at the toxic dose, an evident proinflammatory condition was found, together with the steady increase in the gene expression of interleukin-1α (IL-1α) and interleukin 6 (IL-6). TiO_2_ NPs treatment activated mitogen-activated protein kinase (MAPK) and nuclear factor kappa-light-chain-enhancer of activated B cell (NF-kB) signaling pathway.

Although *in vitro*, this pilot study highlights the adverse effects even of subtoxic dose of TiO_2_ NPs on porcine SCs functionality and viability and, more importantly, set the basis for further *in vivo* studies, especially in chronic exposure at subtoxic dose of TiO_2_ NPs, a condition closer to the human exposure to this nanoagent.

## 2 Materials and Methods

### 2.1 TiO_2_ NPs Synthesis and Characterization

The synthesis of the NPs was performed by sonochemical method using acoustic cavitation (30) at the Laboratory of Acoustic Cavitation of the Department of Physics and Geology of the University of Perugia. The ultrasound generator used was a “Horn Type” working at 20 kHz and 750 W, using a 13-mm titanium probe (Sonics & Materials, Newtown, CT, USA). In particular, anatase phase of TiO_2_ NPs was prepared dissolving 85 ml titanium (IV) butoxyde, (Sigma-Aldrich Co., St. Louis, MO, USA) in 200 ml of pure ethanol (Sigma-Aldrich Co., St. Louis, MO, USA), successively 200 ml of water (Sigma-Aldrich Co., St. Louis, MO, USA) were added dropwise under ultrasound irradiation for 90 min at 50% of amplitude. The obtained precipitate was centrifuged and calcinated at 400°C for 3 h. Chemical-structural characterization of the synthesized NPs was performed by X-ray diffraction (XRD) and electron scanning spectroscopy (SEM). XRD was evaluated at room temperature with the diffractometer Philips X’Pert PRO MPD (Malvern Panalytical Ltd., Royston, UK) operating at 40 kW/40 mA with step size of 0.017° and a step scan of 70 s, using a Cu Kα radiation and X-Celerator detector. SEM analysis was performed using a LEO 1525 Field Emission Scanning Electron Microscope, ZEISS (Jena, Germany). Size distribution of nanoparticle dispersion was performed by dynamic light scattering (DLS) using a Nicomp 380 ZLS spectrometer sorgent 35 mW He–Ne laser at 654 nm and Avalanche PhotoDiode (APD) detector (PSS NICOMP; Santa Barbara, CA, USA), Briefly, starting from a concentration of 1 mg/ml of TiO_2_ NPs dissolved in endotoxin free water (Sigma-Aldrich, St. Louis, MO, USA) to obtain a stock dispersion and sonicated for 30 min to limit the formation of nanoaggregates, dilutions of 10 μg/ml were immediately prepared in different media in order to identify the best experimental conditions for NPs application and more precisely both in SCs culture medium, i.e., Hamster Ovary cells F-12 (HAMF-12), (Euroclone, Milan, Italy), supplemented with 0.166 nM retinoic acid (Sigma-Aldrich Co., St. Louis, MO, USA), 5 ml/500 ml of insulin-transferrin-selenium (ITS), (cat. no. 354352, BD Biosciences, Franklin Lakes, NJ, USA) and, based on literature data (31), Dulbecco’s modified Eagle’s medium (DMEM) (Euroclone, Milan, Italy) supplemented with 0.166 nM retinoic acid (Sigma-Aldrich, St. Louis, MO), 5 ml/500 ml ITS (cat. no. 354352, BD Biosciences, Franklin Lakes, NJ, USA), and 2 mg/ml bovine serum albumin (BSA) (Sigma-Aldrich Co., St. Louis, MO, USA).

### 2.2 SCs Isolation and Characterization

Animal studies were conducted in agreement with the guidelines adopted by the Italian Approved Animal Welfare Assurance (A-3143-01) and the European Communities Council Directive of November 24, 1986 (86/609/EEC). The experimental protocols were approved by the University of Perugia. Danish Duroc prepupertal pigs (15 to 20 days old) underwent bilateral orchidectomy after general anesthesia with ketamine (Ketavet 100; Intervet, Milan, Italy), at a dose of 40 mg/kg, and dexmedetomidine (Dexdomitor, Orion Corporation, Finland), at a dose of 40 g/kg (32), and were used as SC donors. Specifically, pure porcine prepupertal SCs were isolated and characterized, according to previously established methods (33, 34), as reported in the **Supplementary Material**.

### 2.3 Experimental Protocol

NPs working solutions, at a concentration of 5 and 100 µg/ml according to MTT analysis in the **Supplementary Material**, were prepared, administered to SCs for 24 h (acute exposure) and 1, 2, and 3 weeks (chronic exposure) where medium changes were performed every 72 h. The control group was the unexposed SCs (0 TiO_2_ NPs µg/ml). Samples to perform all necessary analyses were collected at each experimental point.

### 2.4 TiO_2_ NPs Uptake by SCs

Cellular uptake of TiO_2_ NPs at 5 and 100 µg/ml was detected with different qualitative and quantitative techniques such as TEM and inductively coupled plasma-optical emission spectrometry (ICP-OES), respectively.

#### 2.4.1 TEM

Briefly, TiO_2_ NP-treated SCs were fixed with 4% glutaraldehyde in 0.1 M sodium cacodylate (NaCaCO) buffer for 30 min and stored at 4°C. Cells were postfixed in 2% osmium tetroxide (OsO_4_) for 2 h and block stained in saturated uranyl acetate. After dehydration, specimens were embedded in epoxy resin (Epon 812), (Sigma-Aldrich Co., St. Louis, MO, USA). Ultrathin sections were cut using a Leica Ultracut R microtome (Leica Microsystem, Austria) with a Diatome knife (Diatome Ltd. CH-2501, Biel, Switzerland) and double stained with uranyl acetate replacement and lead citrate. Sections were viewed and photographed in a Morgagni Series 268D electron microscope (FEI Company, Brno, Czech Republic) equipped with a Megaview III digital camera and Analy-SIS software (Olympus Soft Imaging Solutions).

#### 2.4.2 ICP-OES

TiO_2_ NP-treated SCs were detached by trypsin/ethylenediaminetetraacetic acid (EDTA) (Lonza, Verviers, Belgium) at 37°C for 8 min, to promote the enzymatic reaction. After washing with 1 ml Hank’s balanced salt solution (HBSS) (Sigma-Aldrich Co., St. Louis, MO, USA), samples were centrifuged at 150×*g* for 6 min, the supernatant was removed, the pellets were freeze-dried, and accurately weighed. Samples were dissolved by treatment with 10 ml of a mixture of sulfuric acid (H_2_SO_4_), (97% Sigma-Aldrich Co., St. Louis, MO, USA)/nitric acid (HNO_3_ 70%), (Sigma-Aldrich Co., St. Louis, MO, USA) (2:1). After solubilization, the obtained solutions were diluted with the EDTA solution (1:10) prior Ti^4+^ content determination using a Varian 700-Es series spectrometer (Agilent, Milan, Italy) in triplicate. Calibration was performed diluting a Ti^4+^ standard solution obtaining titanium solutions in the 1–15-μg/ml range. The Ti^4+^ content was expressed per unit weight of freeze-dried TiO_2_ NP-treated SCs and % of the total amount added and the error expressed as SEM.

### 2.5 ROS Detection

Intracellular ROS were measured by treating unexposed and exposed SCs with 50 μM dichlorofluorescein diacetate (DCFH-DA) (Sigma-Aldrich Co., St. Louis, MO, USA) solution in Dulbecco’s phosphate-buffered saline (D-PBS) (Sigma-Aldrich Co., St. Louis, MO, USA) at 37°C for 30 min. Fluorescence was read by using a plate reader (DTX 880 Multimode Detector, Beckman Coulter). Data were normalized for cell viability (MTT assay) and expressed as the percentage of unexposed SCs. The sensitivity of the test was confirmed by adding 30 µM hydrogen peroxide (H_2_O_2_) (30 min) on unexposed SCs as positive control.

### 2.6 Oxidative DNA Damage Quantification by Single-Cell Microgel (Comet) Assay

To evaluate the oxidative DNA damage, unexposed and exposed SCs were processed in the comet assay under alkaline conditions (alkaline unwinding/alkaline electrophoresis, pH >13), basically following the original procedure (35). A positive control consisted in treating SCs with 1 μM 4-nitroquinoline N-oxide (4NQO) (Sigma-Aldrich, Milan, Italy) for 1 h at 37°C (36). At the end of treatments, the cells were washed twice with 5 ml ice-cold D-PBS (Sigma-Aldrich, St. Louis, MO, USA), pH 7.4, and detached with 300 μl of 0.05% trypsin (Invitrogen, Milan, Italy) in 0.02% Na_4_EDTA (Invitrogen, Milan, Italy). After 3 min, trypsinization was stopped by adding 700 μl fetal bovine serum (FBS), (Sigma-Aldrich, St. Louis, MO, USA). Cells were then collected by centrifugation (70×*g*, 8 min, 4°C). Briefly, cell pellets were gently resuspended in 300 μl of 0.7% low-melting point agarose (Sigma-Aldrich, St. Louis, MO, USA) (in Ca^2+/^Mg^2+-^free D-PBS, w/v) at 37°C, layered onto a conventional microscope slide precoated with 1% normal-melting point agarose (in Ca^2+/^Mg^2+^-free D-PBS, w/v), and covered with a coverslip (Knittel-Glaser, Braunschweig, Germany). After brief agarose solidification at 4°C, the coverslip was removed and the slides were immersed in cold, freshly prepared lysing solution (2.5 M sodium chloride (NaCl), 100 mM Na_4_EDTA, (Invitrogen, Milan, Italy); 10 mM tris(hydroxymethyl)aminomethane-hydrochloric acid (Tris-HCl) and sodium hydroxide (NaOH), pH 10, and 1% Triton X-100, (Sigma-Aldrich, Milan, Italy), added just before use, for at least 60 min at 4°C. The slides were then placed in a horizontal electrophoresis box (HU20, Scie-Plas, Cambridge, UK) filled with a freshly prepared solution (10 mM Na_4_EDTA, 300 mM NaOH; pH 13, (Sigma-Aldrich, Milan, Italy). Prior to electrophoresis, the slides were left in the alkaline buffer for 20 min to allow DNA unwinding and expression of alkali-labile damage. Electrophoresis runs were then performed in an ice bath for 20 min by applying an electric field of 34 V (1 V/cm) and adjusting the current to 300 mA (Power Supply PS250, Hybaid, Chesterfield, MO, USA). The microgels were then neutralized with 0.4 M Tris-HCl buffer (pH 7.5), fixed 10 min in ethanol (10 min, Carlo Erba Reagenti, Milan, Italy), allowed to air-dry and stored in slide boxes at room temperature until analysis.

All the steps of the comet assay were conducted in yellow light to prevent the occurrence of additional DNA damage. Immediately before scoring, the air-dried slides were stained with 65 μl of ethidium bromide (EB), (Sigma-Aldrich, St. Louis, MO, USA) 20 mg/ml and covered with a coverslip. The comets in each microgel were analyzed (blind), at ×500magnification with an epi-fluorescent microscope (BX41, Olympus, Tokyo, Japan), equipped with a high sensitivity black and white charge-coupled device (CCD) camera (PE2020, Pulnix, UK), under a 100-W high-pressure mercury lamp (HSH-1030-L, Ushio, Japan), using appropriate optical filters (excitation filter 510–550 nm and emission filter 590 nm). Images were elaborated by Comet Assay III software (Perceptive Instruments, UK). A total of 100 randomly selected comets (50 cells/replicate slides) were evaluated for each experimental point.

### 2.7 RNA Isolation, Reverse Transcription, and Real-Time Reverse Transcriptase-Polymerase Chain Reaction Analyses

Total RNA was isolated with Tri-reagent (Sigma-Aldrich Co., St. Louis, MO, USA) and quantified by reading the optical density at 260 nm. Subsequently, 2.5 μg of total RNA was subjected to reverse transcription (RT Thermo Scientific, Waltham, MA, USA) in a final volume of 20 μl. Real-time reverse transcriptase-polymerase chain reaction (RT-PCR) was performed using 25 ng of the cDNA and SYBR Green master mix (Stratagene, Amsterdam, the Netherlands), as previously described (37).

Gene expression vs. β-actin was evaluated by RT-PCR on a MX3000P Real-Time PCR System (Agilent Technology, Milan, Italy). The sequences of the oligonucleotide primers are listed in **Table 1**. The thermal cycling conditions were 1 cycle at 95°C for 5 min, followed by 45 cycles at 95°C for 20 s and 58°C for 30 s. The data required for carrying out a comparative analysis of gene expression were obtained by means of the 2-(DDCT) method.

**Table 1:** Primer sequences for PCR analyses.

Gene	Forward	Reverse
β-Actin	ATGGTGGGTATGGGTCAGAA	CTTCTCCATGTCGTCCCAGT
AMH	GCGAACTTAGCGTGGACCTG	CTTGGCAGTTGTTGGCTTGATATG
Inhibin B	TGGCTGGATGTCGTCCCAGT	CCGTGTGGAAGGATGAGG
SOD1	TCGGGAGACCATTCCATCAT	ACCTCTGCCCAAGTCATCT
HO-1	CTGGTGATGGCGTCCTTGTA	TTGTTGTGCTCAATCTCCTCCT
GHSPx	CGAGAAGTGTGAGGTGAATGG	GCGGAGGAAGGCGAAGAG
LDH A	GCACCCTGAATTAGGCACTGATG	ATAAGCACTGTCCACCACCTGTT
IL-1α	GCAAGTTCCTGTGACTCTAAGAAT	TTTGGATGGGCGGCTGAAT
IL-6	AATGCTCTTCACCTCTCC	TCACACTTCTCATACTTCTCA
TGF-β1	GCCCTGGACACCAACTATTGC	GCTGCACTTGCAGGAGCGCAC
IDO	ATGAAGGCGTTTGGGACACC	GAGGAATCCAGCAGCAGAGC

AMH, anti-Müllerian hormone, SOD1, superoxide dismutase 1, HO-1, heme-oxygenase 1; GHSPx, gluthatione peroxidase; LDH-A, lactate deidrogenase A; IL-1α, interleukin-1α; IL-6, interleukin-6; TGF-β1, transforming growth factor β1; IDO, indoleamine 2,3-dioxygenase.

### 2.8 Elisa Assay

Aliquots of the culture media from all the experimental groups were collected, stored at −20°C for the assessment of AMH (AMH Gen II ELISA, Beckman Coulter; intraassay CV = 3.89%; interassay CV = 5.77%) and inhibin B (inhibin B Gen II ELISA, Beckman Coulter, Webster, TX, USA; intraassay CV = 2.81%; interassay CV = 4.33%) secretion as previously described (34).

### 2.9 Protein Extraction and Western Blot Analysis

Total protein extracts were prepared by lysing the cells in 100 μl of radioimmunoprecipitation assay lysis buffer (RIPA buffer), (Santa Cruz Biotechnology Inc., Santa Cruz, CA, USA) as previously described (38).

After centrifuging the mixture at 1,000×*g* (Eppendorf, NY, USA) for 10 min, the supernatant was collected, and total protein content was determined by the Bradford method (39). Sample aliquots were stored at −20°C for WB analysis. The cell extracts were separated by 4%–12% sodium dodecyl sulfate-polyacrylamide gel electrophoresis (SDS-PAGE), and equal amounts of protein (70 μg protein/lane) were run and then blotted on nitrocellulose membranes (Bio-Rad, Hercules, CA, USA). The membranes were incubated overnight in a buffer containing 10 mM TRIS (Sigma-Aldrich Co., St. Louis, MO, USA), 0.5 M NaCl (Sigma-Aldrich Co., St. Louis, MO, USA), 1% (v/v) Tween 20 (Sigma-Aldrich Co., St. Louis, MO, USA), rabbit antiextracellular signal-regulated kinases 1–2 (ERK1–2) (1:2000; Millipore, Burlington, MA, USA), mouse antiphospho-ERK1-2 (1:100; Millipore, Burlington, MA, USA), rabbit anti-c-Jun N-terminal kinases (JNK) (1:1000; Millipore, Burlington, MA, USA), rabbit antiphospho-JNK (1:500; Millipore, Burlington, MA, USA), rabbit antiposphop38 (1:2000; Millipore, Burlington, MA, USA), mouse anti-p38 (1:2000; Millipore, Burlington, MA, USA), antiserine/threonine protein kinase (Akt) (1:100; Cell Signaling, Danvers, MA, USA), rabbit antiphospho-Akt (1:1000; Cell Signaling, Danvers, MA, USA), rabbit antiphospho-NF-kB p65 antibody (1:1000; AbCam, Cambridge, UK), rabbit anti-NF-kB p65 antibody (1:1000; AbCam, Cambridge, UK), rabbit anticaspase 3 antibody (1:1000; Cell Signaling, Danvers, MA, USA), and mouse anti-GAPDH (1:5000; Sigma-Aldrich Co., St. Louis, MO, USA) primary antibodies.

Primary antibody binding was then detected by incubating membranes for an additional 60 min in a buffer containing horseradish peroxidase conjugated antirabbit (1:5000; Sigma-Aldrich Co., St. Louis, MO, USA) and/or antimouse (1:5000; Santa Cruz Biotechnology Inc.) IgG secondary antibodies. The bands were detected by enhanced chemiluminescence and acquired by ChemiDoc imaging System (Bio-Rad, Hercules, CA, USA).

### 2.10 Statistical Analysis

Normality analysis was performed by Shapiro-Wilk test, and statistical comparisons were analyzed using one-way ANOVA followed by Tukey’s HSD *post-hoc* test (SigmaStat 4.0 software, Systat Software Inc., CA, USA). Values were reported as the means ± SEM of three independent experiments, each performed in triplicate. Differences were considered statistically significant at ^*^ *p* < 0.05 and ^**^ *p* < 0.001 compared with unexposed SCs and ^#^ *p* < 005 with respect to 5 μg/ml of TiO_2_ NPs.

## 3 Results

### 3.1 Characterization and Size Distribution of TiO_2_ NPs

**Figure 1:**
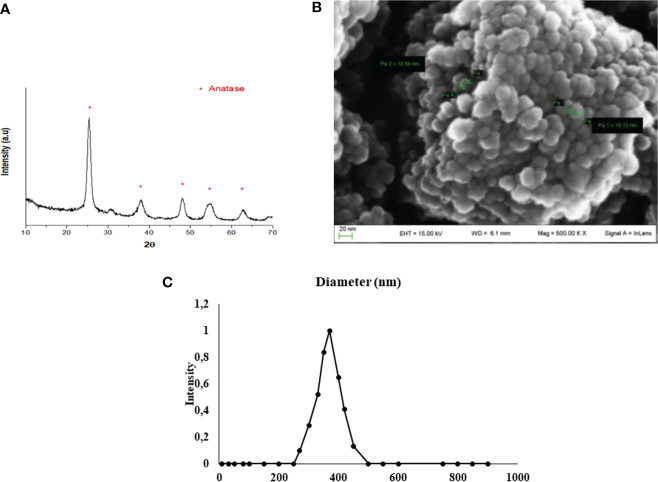
TiO_2_ NPs characterization. **(A)** XRD analysis: diffractogram confirmed the correct synthesis of TiO_2_ NPs (JCPDS 00-001-0562) and * represents the peaks of TiO_2_ NPs in anatase form. **(B)** SEM analysis: the mean size distribution of TiO_2_ NPs in dry form was 20 ± 5 nm; image confirmed that TiO_2_ NPs tend to form aggregates of submicrometric dimensions. **(C)** DLS analysis: mean hydrodynamic diameter of TiO_2_ NPs at 10 µg/ml in culture medium.

XRD analysis, as a methodology used to determine the structure of inorganic compounds, confirmed the synthesis of TiO_2_ NPs. In fact, the resulting diffractogram was characteristic of titanium dioxide crystals in anatase form (JCPDS 00-001-0562) (**Figure 1A**). https://doi.org/10.5061/dryad.7d7wm37wf.

SEM evaluation, through a nondestructive technique, allowed to perform morphological and measurement investigation of TiO_2_ NP surfaces. A representative SEM image of TiO_2_ NPs in dry form is showed in **Figure 1B**, and the mean size distribution reports values of 20 ± 5 nm diameter, calculated by measuring over 100 particles in random fields of view. Results showed that TiO_2_ NPs tended to form aggregates of submicrometric dimensions.

DLS analysis, being able to measure the hydrodynamic diameter of NPs dispersed in a liquid, confirmed some aggregation of TiO_2_ NPs in suspension. The mean hydrodynamic diameter of TiO_2_ NPs mainly distributed in a range of 100–300 nm (**Figure 1C**). Additional information on NP suspension setups can be found in **Supplementary Material**.

### 3.2 Light Microscopy Analysis Revealed That TiO_2_ NPs Caused Significant Morphological Alterations at the Toxic Dose

**Figure 2:**
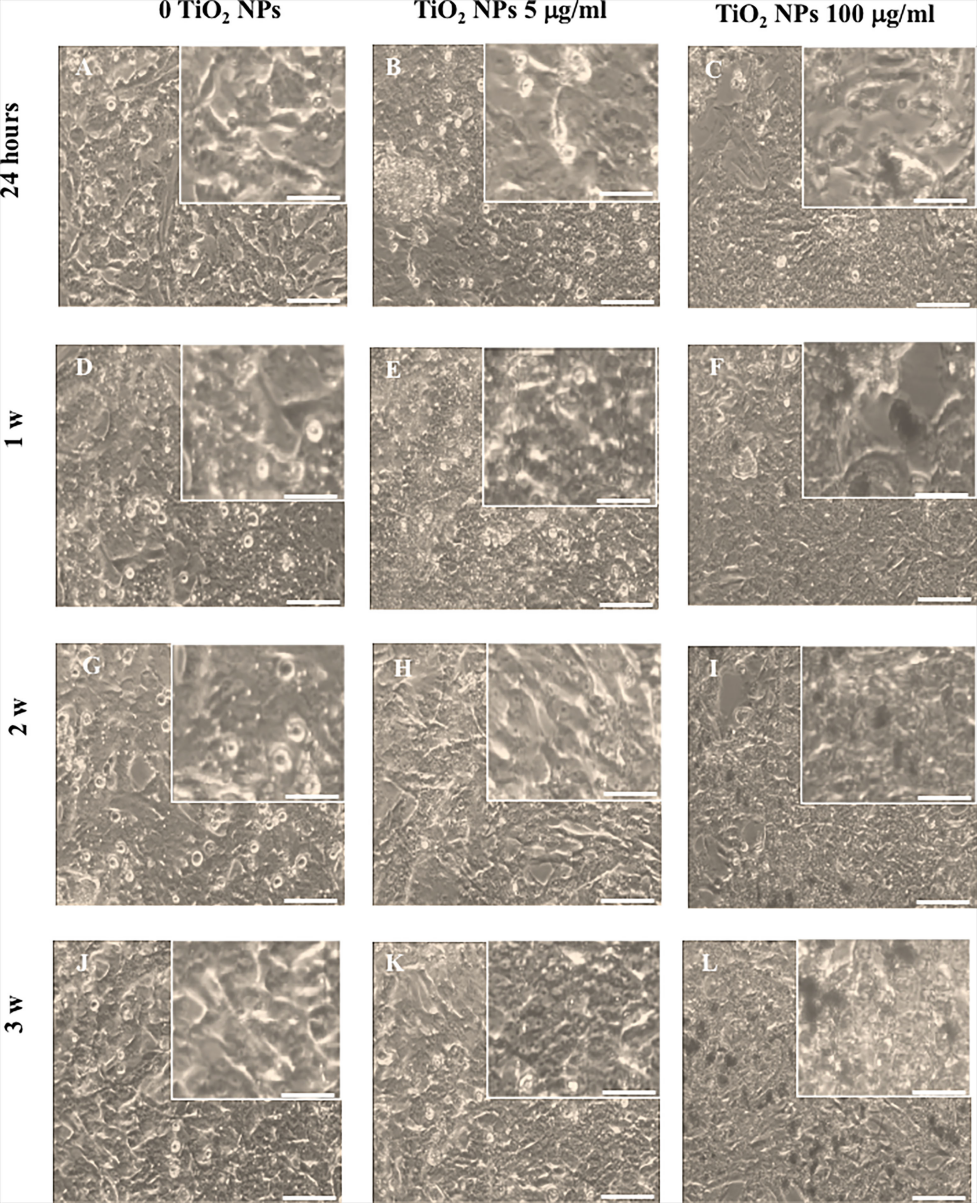
Morphological characterization. Light microscope of unexposed (0 TiO_2_ NPs) SCs **(A, D, G, J)**, TiO_2_ NPs 5 **(B, E, H, K)**, and 100 µg/ml **(C, F, I, L)** exposed SCs at 24 h **(A–C)** and 1 **(D–F)**, 2 **(G–I)**, and 3 weeks **(L–N)**. The inserts show in detail the morphological characteristics of the respective images. The scale bar corresponds to 200 µm for **(A–N)** and 60 µm for the corresponding insets. The images are representative of three separate experiments.

Light microscopy analysis was used to monitor over the course of the experiment any morphological changes in the SCs treated with the two TiO_2_ NP concentrations with respect to the unexposed monolayer (**Figures 2A, D, G, J**). Morphological analysis revealed that SCs treated with 5 µg/ml TiO_2_ NPs did not undergo substantial changes compared with the untreated monolayer at all times of exposure. In fact, cells exposed to TiO_2_ NPs maintained the typical squamous shape of epithelial cells with vacuoles containing lipid hormones, well evident and abundantly distributed in the cell surface (**Figures 2B, E, H, K** **)**. Conversely, SCs treated with 100 µg/ml TiO_2_ NPs showed an evident reduction either of the cell number that constituted the monolayer (**Figure 2C**; **Supplementary Figure 2S**) and changes in the morphology of the remaining cells that showed a spindle-like shape, with very elongated cytoplasmic protrusions, typical of a cellular stress-associated condition by the end of the first week (**Figure 2F**) that progressively increased until the end of the third week (**Figures 2I, L** **)**.

### 3.3 Qualitative (TEM) and Quantitative Analyses (ICP-OES) Confirmed the Uptake of TiO_2_ NPs by SCs

**Figure 3:**
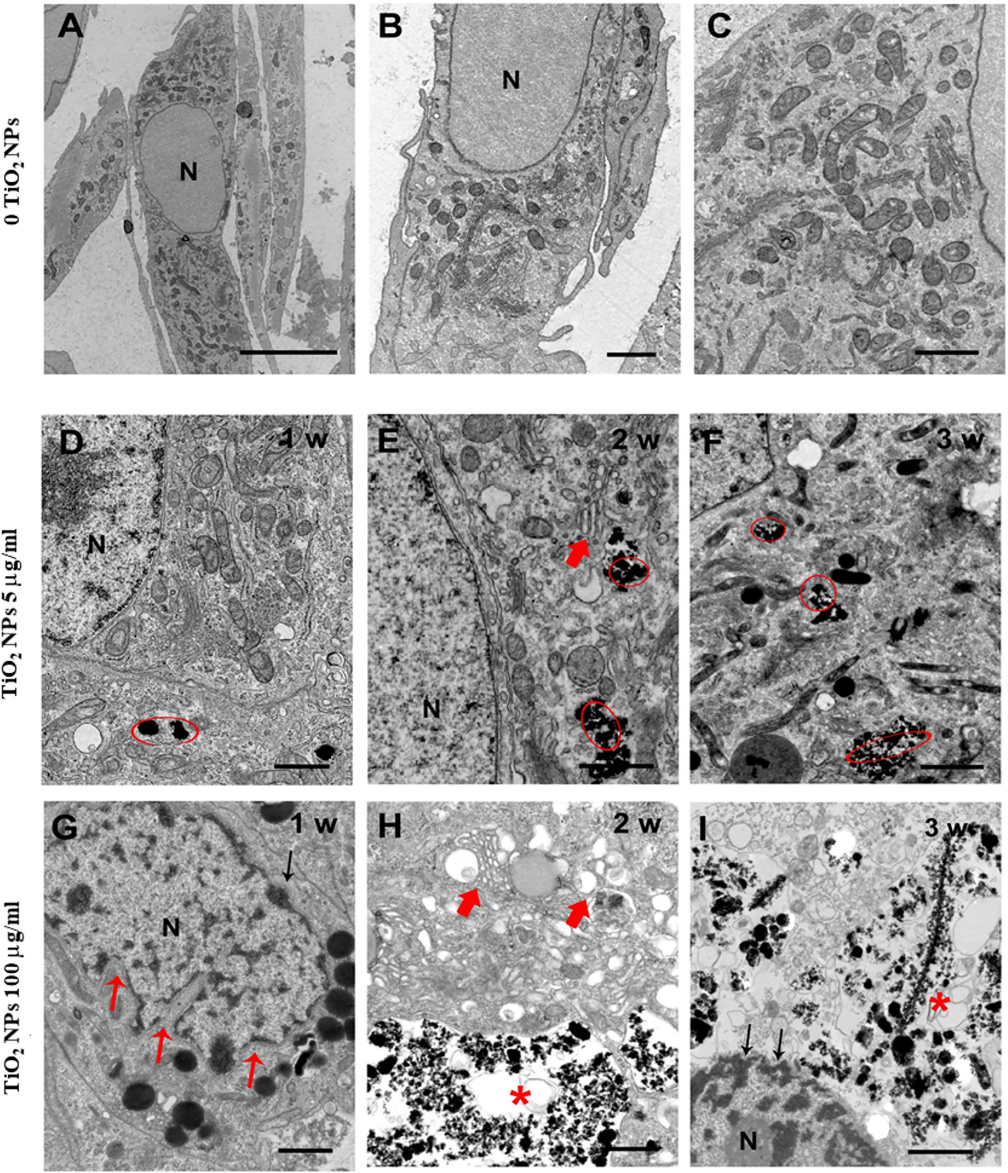
Representative TEM images of unexposed SCs **(A–C)** and after treatment with TiO_2_ NPs at 5 and 100 µg/ml for 1, 2, and 3 weeks of exposure **(D–I)**. **(A–C)** Elongated oval or spindle-shaped SCs containing quite large nuclei (N) and abundant mitochondria [better visible at higher magnification in **(C)**]. **(D–I)** SCs after treatment with TiO_2_ NPs at 5 µg/ml **(D–F)** and 100 µg/ml/L **(G–I)** for 1, 2, and 3 weeks of exposure (chronic). Red circles and asterisks indicate TiO_2_ NPs deposition. Small red arrows indicate nuclear membrane invaginations. Large red arrows indicate enlarged endoplasmic reticulum. Black arrows in **(I)** point to chromatin marginalization. The scale bar corresponds to 5 µm for **(A)**, 2 µm for the **(B)**, and 1 µm for **(C–I)**.

TEM has been used both to verify the uptake of NPs and to assess any ultrastructural changes in SCs as a result of this interaction. TEM analysis of unexposed SCs outlined a typical architecture characterized by oval/elongated spindle-shaped morphology containing a quite large nucleus surrounded by abundant mitochondria and numerous aggregates of stacks of parallel and flat cisternae of endoplasmic reticulum membranes (**Figures 3A–C**). Although we did not notice substantial and/or severe ultrastructural alterations in SCs treated with 5 µg/ml TiO_2_ NPs for 1 and 2 weeks (**Figures 3D, E**), in all the other exposure conditions (5 µg/ml for 3 weeks and 100 µg/ml for 1, 2, and 3 weeks), (**Figures 3F, I**) SCs showed several ultrastructural changes. Specifically, we noted a high percentage of severely damaged SCs, presenting different features of cell stress such as deeply invaginated and shrunk nuclei (**Figure 3G**), disorder/marginalization of chromatin components (**Figures 3G, I** **)**, swollen very fragmented, or almost completely missing endoplasmic reticulum membranes (**Figures 3E, H** **)**. In addition, mitochondrial morphology was severely affected (**Figure 3F**) and mitochondria number dramatically decreased. Finally, we noted the presence of several large vacuoles (**Figures 3H, I** **)** probably as a result of apoptotic mitochondria and/or enlarged endoplasmic reticulum and/or increased frequency of lipid droplets, https://doi.org/10.5061/dryad.7d7wm37wf.

**Figure 4:**
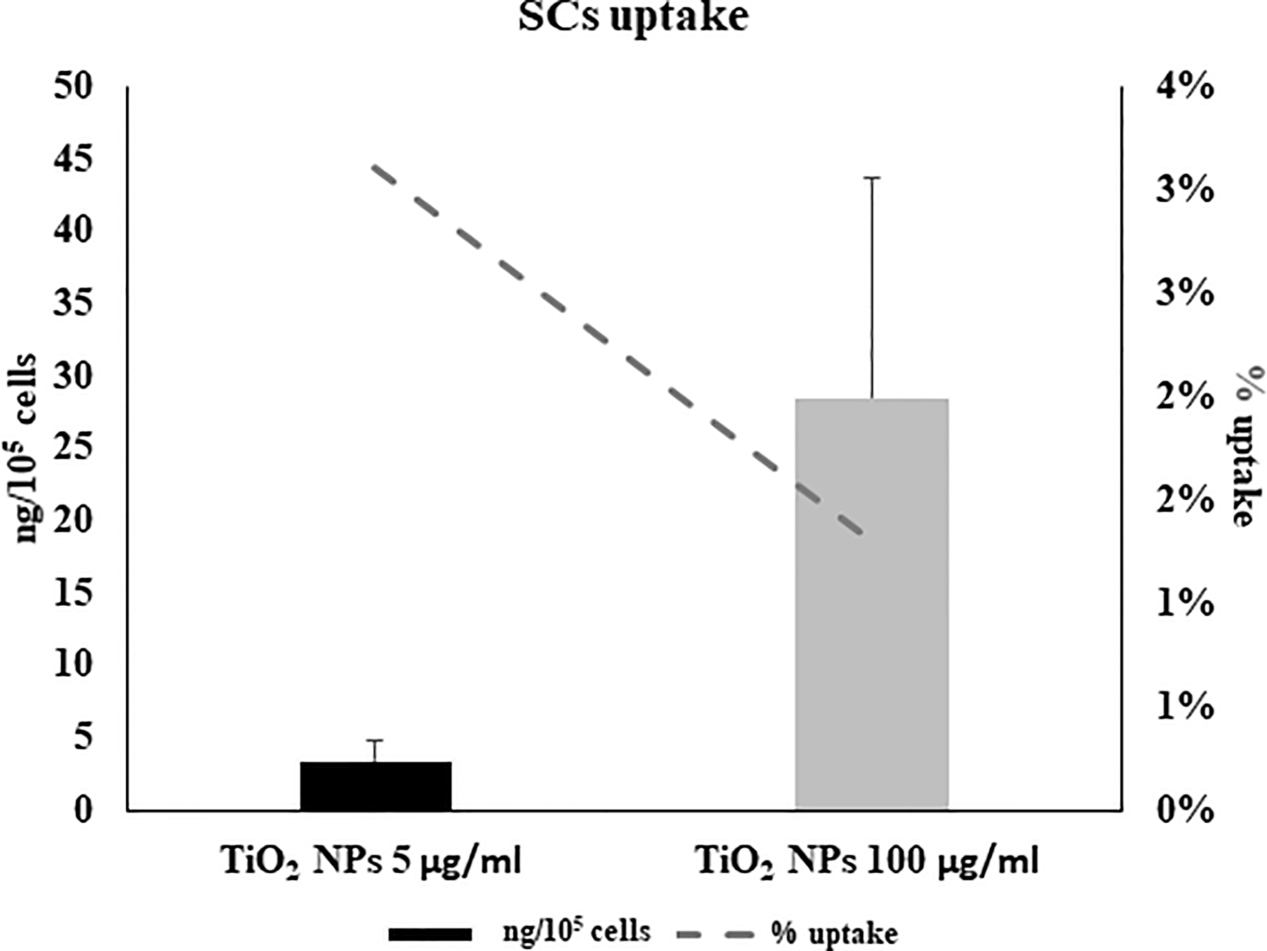
Cellular uptake by inductively coupled plasma-optical emission spectrometry (ICP-OES). Percentage of internalized nanoparticles (dotted grey line) and amount of metal adsorbed per cell number (expressed as ng/10^5^) in SCs 5 h of incubation with TiO_2_ NPs 5 (black bar) and 100 µg/ml (grey bar) evaluated by ICP-OES analysis. Data represented as mean ± SEM of three independent experiments, each are performed in triplicate.

ICP-OES was used to quantify the uptake of NPs expressed as the percentage of internalized NPs and the amount of metal adsorbed per cell number (expressed as ng/10^5^), at each concentration, after 5 h of treatment (**Figure 4**). At the lowest concentration of 5 μg/ml, a 3% higher uptake rate was detected than at the 100-μg/ml concentration (1.3%) (**Figure 4**, dotted line). In contrast, the amount absorbed in ng/10^5^ cells was greater at the highest concentration (28.6 ± 15.01 vs. 3.46 ± 1.3, **Figure 4**, black bars). This difference is likely attributable to gradual saturation of the particles within the cells with a progressive slowing of uptake as the concentration of NPs increases.

### 3.4 TiO_2_ NPs Exposure Induced Apoptosis in SCs

We assessed, by Western blotting analysis, the involvement of caspase-3 pathway that is partially or totally responsible for the proteolytic cleavage of many key proteins, such as the nuclear enzyme poly (ADP-ribose) polymerase (PARP) (40). Activation of caspase-3 requires proteolytic processing of its inactive zymogen (p35) into activated p19 and p17 fragments (40).

We demonstrated that NPs exposure, at each concentration, induced the activation of caspase-3 at the first and second week, with the cleavage of p35 into the p19 kDa active fragment, to decrease at the third week, most likely due to the progress toward the final degradation phase of apoptosis (41).

**Figure 5:**
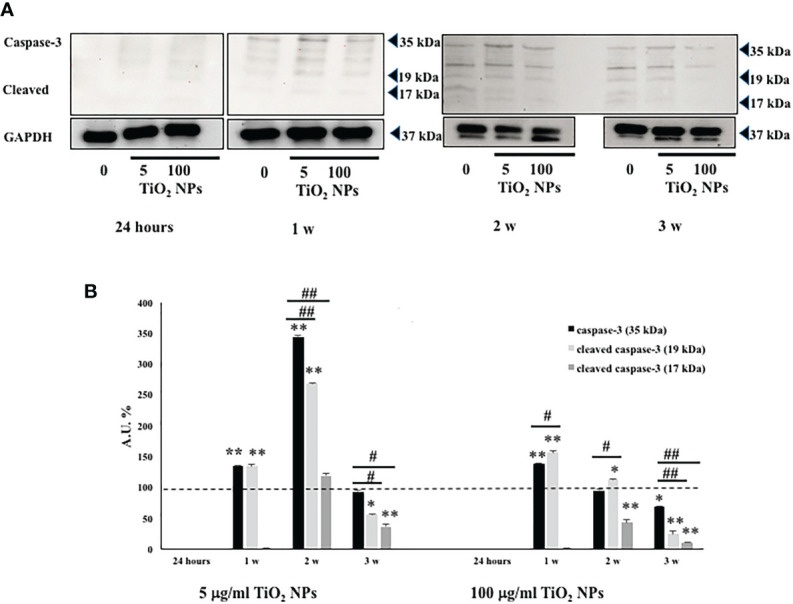
WB analysis. **(A)** Immunoblots of caspase-3 p35, p19, and p17 in SCs at 24 h and 1, 2, and 3 weeks of incubation with TiO_2_ NPs at 5 and 100 µg/ml. **(B)** Densitometric analysis of the protein bands of caspase-3 p35 (black bar), p19 (light grey bar), and p17 (dark grey bar) in SCs at 24 h and 1, 2, and 3 weeks of incubation with TiO_2_ NPs 5 and 100 µg/ml. Data represent the mean ± SEM (^*^ *p* < 0.05,^**^ *p* < 0.001 vs. unexposed SCs (black dotted line) and ^#^ *p* < 0.05, ^##^ *p* < 0.001 vs. p35 of three independent experiments, each performed in triplicate).

Only at the toxic dose, we observed a statistical increase of active p19 with respect to the inactive p35 fragments, expression of a more prominent apoptotic process, as highlighted by TEM analysis (**Figures 5A, B**, ^#^ *p* < 0.05, ^##^ *p* < 0.001 vs. p35).

### 3.5 Effects of TiO_2_ NPs on Intracellular ROS Production and DNA Damage

We evaluated the intracellular production of ROS and oxidative DNA damage, as the most important mechanisms activated by nanomaterial.

**Figure 6:**
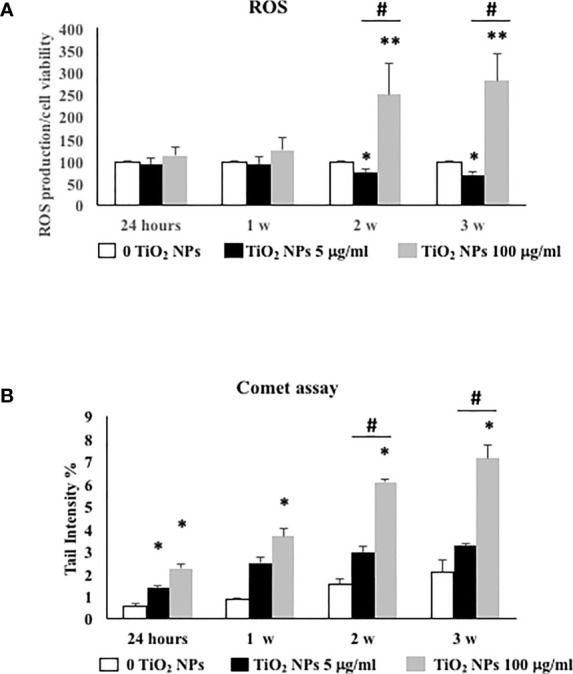
ROS production and DNA damage under TiO_2_ NPs treatment in SCs. **(A)** Total intracellular ROS production in SCs exposed to TiO_2_ NPs 5 (black bar) and 100 µg/ml (grey bar) for 24 h and 1, 2, and 3 weeks. Data represent the mean ± SEM (^*^ *p* < 0.05, ^**^ *p* < 0.001, with respect to unexposed SCs (black dotted line) and ^#^ *p* < 0.05 vs. 5 μg/ml of TiO_2_ NPs of three independent experiments, *n* = 8). **(B)** DNA damage expressed as tail intensity % in unexposed SCs (white bar) and exposed to TiO_2_ NPs 5 (black bar) and 100 µg/ml (grey bar) for 24 h and 1, 2, and 3 weeks. Data represent the mean ± SEM (^*^ *p* < 0.05, ^**^ *p* < 0.001 vs. unexposed SCs and ^#^ *p* < 0.05 vs. 5 μg/ml of TiO_2_ NPs of three independent experiments, *n* = 8).

As shown in **Figure 6A**, the dose of 5 µg/ml TiO_2_ NPs did not affect ROS intracellular level at 24 h and 1 week postexposure. On the contrary, at 2 and 3 weeks posttreatment, ROS level significantly decreased with respect to unexposed SCs (set as 100 in the graphs and represented as a black dotted line, **Figure 6A**, ^*^ *p* < 0.05). Conversely, 100 µg/ml TiO_2_ NPs induced a significant increase of intracellular ROS amounts at the second and third week posttreatment with respect to unexposed SCs (set as 100 in the graphs and represented as a black dotted line, **Figure 6A**, ^*^ *p* < 0.05 and ^**^ *p* < 0.001) accompanied by a significant increase at the third week compared with 5 μg/ml of TiO_2_ NPs, expression of oxidative stress onset with increasing concentration (**Figure 6A**, ^#^ *p* < 0.05 vs. 5 μg/ml of TiO_2_ NPs). As expected, H_2_O_2_ (positive control) induced a significant increase in ROS intracellular levels (data not shown).

The levels of oxidative DNA damage induced by TiO_2_ NPs were measured as the % of DNA in tail by the alkaline comet assay over time, after acute (24 h) and chronic exposures (from 1 to 3 weeks) to 5 and 100 µg/ml of TiO_2_ NPs. At the dose of 5 μg/ml, the oxidative DNA damage was detected after 24 h of acute exposure (**Figure 6B**, ^*^ *p* < 0.05). The follow-up study carried out after 1 and 3 weeks of chronic exposure exhibited an increase that however did not result in a significant difference compared with unexposed SCs (**Figure 6B**). Instead, the dose of 100 μg/ml induced a significant increase over time and until the end of treatment with respect to the unexposed SCs and from the second week compared with 5 μg/ml of TiO_2_ NPs, as demonstrated by the increased oxidative DNA damage with increasing concentration (**Figure 6B**, ^##^ *p* < 0.001 vs. 5 μg/ml of TiO_2_ NPs).

### 3.6 TiO_2_ NPs Exposure Negatively Affected SCs Functionality

The effects of TiO_2_ NPs exposure on SCs functional competence were studied through the evaluation of AMH and inhibin B gene expression and protein secretion, specific and important markers of SCs functionality.

**Figure 7:**
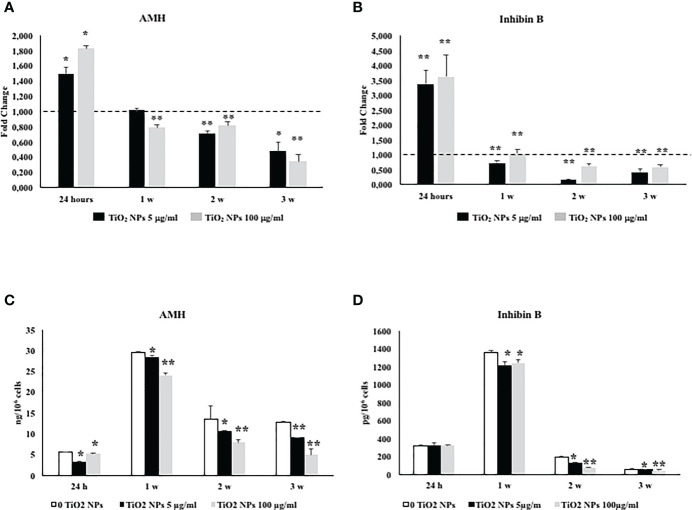
Real-time PCR analysis of SCs functionality. Gene expression of AMH **(A)**, and inhibin B **(B)** in SCs at 24 h and 1, 2, and 3 weeks of incubation with TiO_2_ NPs 5 (black bar) and 100 µg/ml (grey bar). ELISA assay of **(C)** AMH and **(D)** inhibin B secretion in SCs at 24 h and 1, 2, and 3 weeks of incubation with TiO_2_ NPs 5 (black bar) and 100 µg/ml (grey bar). Data represent the mean ± SEM (^*^ *p* < 0.05 and ^**^ *p* < 0.001 vs. unexposed SCs (0 TiO_2_ NPs, black dotted line) of three independent experiments, each performed in triplicate).

Exposure of SCs toTiO_2_ NPs induced a significant increase in AMH and inhibin B gene expression at 24 h followed by a significant decrease after 1 week up to the third week, at both concentrations with respect to unexposed SCs (**Figures 7A, B**, ^*^ *p* < 0.05 and ^**^ *p* < 0.001 vs. unexposed SCs, set as 1 in the graphs and represented as a black dotted line). AMH secretion was significantly and steadily decreased at 24 h throughout the duration of the experiment, whereas inhibin secretion showed a significant decrease starting from the first week onwards (**Figures 7C, D**, ^*^ *p* < 0.05 and ^**^ *p* < 0.001 vs. unexposed SCs).

### 3.7 TiO_2_ NPs Exposure Activated an Antioxidant Response and Damaged the Membrane Integrity

To investigate the role and effectiveness of phase II detoxification enzymes against ROS production, we evaluated SOD, glutathione peroxidase (GHSPx), and HO-1 gene expression. In addition, we studied lactate dehydrogenase-A (LDH-A) gene expression as a well-established and reliable indicator of cellular toxicity and plasma membrane damage.

**Figure 8:**
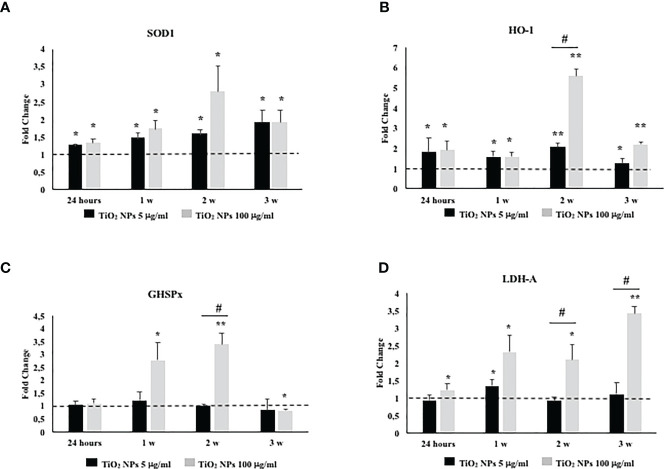
Real-time PCR analysis of SCs antioxidant and metabolic enzymes. Gene expression of SOD1 **(A)**, HO-1 **(B)**, GHSPx **(C)**, and LDH A **(D)** in SCs at 24 h and 1, 2, and 3 weeks of incubation with TiO_2_ NPs 5 (black bar) and 100 µg/ml (grey bar). Data represent the mean ± SEM (^*^ *p* < 0.05, ^**^ *p* < 0.001 vs. unexposed SCs (black dotted line) and ^#^ *p* < 0.05 vs. 5 μg/ml of TiO_2_ NPs of three independent experiments, each performed in triplicate).

The gene expression of SOD1 and HO-1 increased throughout the treatment at both TiO_2_ NP concentrations (**Figures 8A, B**, ^*^ *p* < 0.05, ^**^ *p* < 0.001 vs. unexposed SCs, set as 1 in the graphs and represented as a black dotted line). The gene expression of GHSPx increased at the toxic dose of 100 µg/ml after the first and second week of exposure to undergo a significant reduction at the third week of the treatment (**Figure 8C**, ^*^ *p* < 0.05 and ^**^ *p* < 0.001 vs. unexposed SCs, set as 1 in the graphs and represented as a black dotted line). In particular, only at the second week, the dose of 100 µg/ml of TiO_2_ NPs showed a significant increase of HO-1 and GHSPx gene expression with respect to the dose of 5 µg/ml of TiO_2_ NPs (**Figures 8B, C**, ^#^ *p* < 0.05 vs. 5 μg/ml of TiO_2_ NPs).

A significant increase in LDH-A mRNA expression was observed at 100 µg/ml of TiO_2_ NPs throughout the whole experiment with respect to the unexposed SCs. In addition, a significant increase was observed from the second week even when compared with the dose of 5 µg/ml of TiO_2_ NPs (**Figure 8D**, ^*^ *p* < 0.05 and ^**^ *p* < 0.001 vs. unexposed SCs, set as 1 in the graphs and represented as a black dotted line; ^#^ *p* < 0.05 vs. 5 μg/ml of TiO_2_ NPs).

### 3.8 TiO_2_ NPs Exposure Stimulated Proinflammatory and Immunomodulatory Responses

To assess if TiO_2_ NPs treatment could activate an inflammatory response, we evaluated the gene expression either of proinflammatory cytokines, such as, IL-1α, IL-6, and typical immunomodulatory factors expressed by SCs as transforming growth factor-β (TGF-β) and indoleamine 2,3-dioxygenase (IDO) (42).

**Figure 9:**
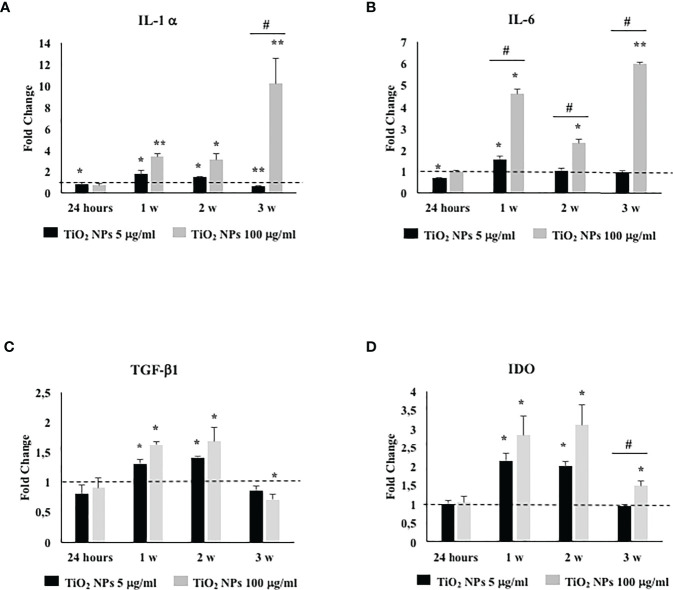
Real-time PCR analysis of SCs proinflammatory and immunomodulatory response. Gene expression of IL-1α **(A)**, IL-6 **(B)**, TGF-β1 **(C)**, and IDO **(D)** in SCs at 24 h and 1, 2, and 3 weeks of incubation with TiO_2_ NPs 5 (black bar) and 100 µg/ml (grey bar). Data represent the mean ± SEM (^*^ *p* < 0.05, ^**^ *p* < 0.001 vs. unexposed SCs (black dotted line) and ^#^ *p* < 0.05 vs. 5 µg/ml of TiO_2_ NPs of three independent experiments, each performed in triplicate).

The gene expression of IL-1α and IL-6 showed a significant increase at the first and second week of treatment with the subtoxic dose meanwhile at the toxic dose increased starting from the first week postexposure up to the end of the experiment (**Figures 9A, B**, ^*^ *p* < 0.05, ^**^ *p* < 0.001 vs. unexposed SCs, set as 1 in the graphs and represented as a black dotted line). Moreover, IL-1α gene expression showed a significant increase compared with the dose of 5 µg/ml of TiO_2_ NPs only at the third week. IL-6 gene expression, at the highest dose, showed a statistical significant increase from the first week throughout the whole experiment with respect to the lowest dose as expression of an enhanced inflammatory state was probably linked to the increasing TiO_2_ NP concentrations (**Figures 9A, B**, ^#^ *p* < 0.05 vs. 5 µg/ml of TiO_2_ NPs).

The gene expression of TGF-β1 and IDO, at the subtoxic dose, showed an upregulation only at the first and second week respect to the unexposed SCs. At the toxic dose, the gene expression of TGF-β1 was upregulated at the first and second week; meanwhile, IDO showed an upregulation from the first to the third week with respect to the unexposed SCs (set as 1 in the graphs and represented as a black dotted line) and exhibited a significant increase compared with the lowest dose only at the third week (**Figures 9C, D**, ^*^ *p* < 0.05, ^**^ *p* < 0.001 vs. unexposed SCs; ^#^ *p* < 0.05 vs. 5 µg/ml of TiO_2_ NPs).

### 3.9 TiO_2_ NPs Treatment Activated MAPK and NF-kB Signaling Pathway

**Figure 10:**
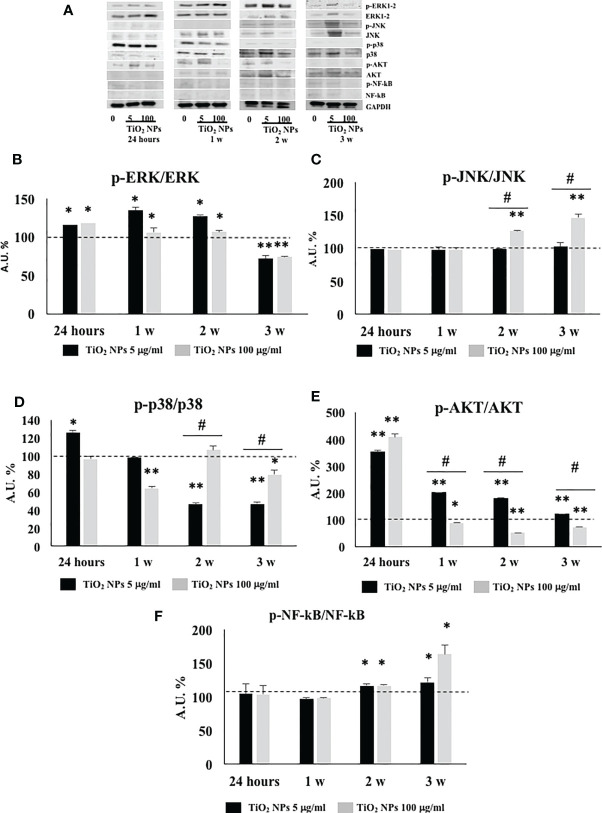
WB analysis. **(A)** Immunoblots of phosphoERK1-2/ERK1-2, phosphoJNK/JNK, phosphop38/p38, phosphoAKT/AKT, posphoNF-kB p65/NF-kB, and GAPDH in SCs 24 h and 1, 2, and 3 weeks of incubation with TiO_2_ NPs 5 and 100 µg/ml. Densitometric analysis of the protein bands of phosphoERK1-2/ERK1-2 **(B)**, phosphoJNK/JNK **(C)**, phosphop38/p38 **(D)**, phosphoAKT/AKT **(E)**, and phosphoNF-kB p65/NF-kB **(F)** in SCs 24 h and 1, 2, and 3 weeks of incubation with TiO_2_ NPs 5 (black bar) and 100 µg/ml (grey bar). Data represent the mean ± SEM (^*^ *p* < 0.05, ^**^ *p* < 0.001 vs. unexposed SCs (black dotted line) and ^#^ *p* < 0.05 vs. 5 µg/ml of TiO_2_ NPs of three independent experiments, each performed in triplicate).

We performed Western blotting analysis to investigate the involvement and timing of activation of different MAPK family members (ERK1/2, JNK, and p38) and NF-kB signaling pathway after TiO_2_ NPs exposure (**Figure 10A**).

The phosphorylation ratio of ERK1/2 showed a significant increase (**Figure 10B**, ^*^ *p* < 0.05, ^**^ *p* < 0.001 vs. unexposed SCs, set as 100 in the graphs and represented as a black dotted line) at 24 h postexposure that persisted until the second week posttreatment, to be downregulated at the third week postexposure, at both concentrations.

The phosphorylation ratio of JNK remained unaltered at both concentrations, at 24 h and 1 week postexposure, but showed a significant increase from the second and third week only at the highest concentration of 100 µg/ml (**Figure 10C**, ^**^ *p* < 0.001 vs. unexposed SCs, set as 100 in the graphs and represented as a black dotted line). In addition, the highest dose exhibited a significant increase starting from the second week in comparison with the lowest dose of NPs (**Figure 10C**, ^#^ *p* < 0.05 vs. 5 µg/ml of TiO_2_ NPs).

The phosphorylation ratio of p38 showed a significant increase at 24 h, at the lowest dose of 5 µg/ml but was followed by a progressive decrease that became significant at the second and third week after exposure (**Figure 10D**, ^*^ *p* < 0.05, ^**^ *p* < 0.001 vs. unexposed SCs, set as 100 in the graphs and represented as a black dotted line). At the highest concentration of 100 µg/ml, p38-dependent pathway showed a significant increase with respect to the lowest dose from the second week (**Figure 10D**, ^*^ *p* < 0.05, ^**^ *p* < 0.001 vs. unexposed SCs, set as 100 in the graphs and represented as a black dotted line; ^#^ *p* < 0.05 vs. 5 µg/ml of TiO_2_ NPs).

The phosphorylation ratio of AKT showed a significant increase throughout the whole experiment at the subtoxic dose but with an inverse time-dependent relationship (**Figure 10E**, ^*^ *p* < 0.05, ^**^ *p* < 0.001 vs. unexposed SCs (set as 100 in the graphs and represented as a black dotted line). At the toxic dose, a significant increase at 24 h was followed by a significant decrease over time (**Figure 10E**, ^*^ *p* < 0.05, ^**^ *p* < 0.001 vs. unexposed SCs (set as 100 in the graphs and represented as a black dotted line). In comparison with the lowest dose, the toxic dose showed a significant decrease from the first week (**Figure 10E**, ^#^ *p* < 0.05 vs. 5 µg/ml of TiO_2_ NPs).

Finally, the phosphorylation ratio of p-NF-kB remained unchanged following acute exposure and at 1 week at both 5 and 100 µg/ml TiO_2_ NPs. Subsequently, it showed a significant increase after the second and third week posttreatment at both concentrations (**Figure 10F**, ^*^ *p* < 0.05, ^**^ *p* < 0.001 vs. unexposed SCs, set as 100 in the graphs and represented as a black dotted line).

## 4 Discussion

In the present study, we evaluated for the first time, to our knowledge, the effects of TiO_2_ NPs on SCs from porcine prepubertal pig testes, an experimental animal model sharing significant physiological similarity with humans, thus providing novel results of a major translational importance.

It is important to note that primary SCs cultures have been recognized as a valuable *in vitro* system to study the effects of heavy metals and toxic substances (43–45).

To date, several *in vivo* and *in vitro* studies, mainly of mouse and rat origin, have evaluated the toxicological effects of TiO_2_ NPs (19–24, 26, 27). Although interesting, the results are often contradictory, suggesting that the potential cell damage is affected by many factors including cell type, nanoparticle size, and dispersion protocols (46). In particular, the dimensions of TiO_2_ NPs are critical from the toxicity point of view, because, as it is well known, NPs are more toxic and reactive than conventional TiO_2_ particles (47).

Aggregates of NPs have been observed in Leydig-Sertoli cells and spermatids when mice were prenatally exposed to anatase TiO_2_ NPs *via* subcutaneous injections (48, 49). In our study, several dispersion protocols were executed, followed by DLS analysis, in order to establish the best experimental condition, where the best choice resulted in DMEM with the addition of 2% BSA to obtain the smallest and stable nanoaggregates as reported in **Supplementary Materials**.

In particular, TiO_2_ NP-treated SCs at 5 µg/ml apparently showed no morphological changes throughout the experimental treatment, but a deeper evaluation of this apparent health condition by MTT analysis revealed its cytotoxic effect under chronic exposure, with a 20% decrease in metabolically active cells (**Supplementary Material**), accompanied by important structural abnormalities revealed by TEM analysis at the third week.

At the concentration of 100 µg/ml TiO_2_ NPs, SCs showed a spindle-like shape, with very elongated cytoplasmic protrusions, typical of a cellular stress-associated condition, by the end of the first week that progressively increased until the end of the third week, accompanied by a steady decrease around 50% of metabolically active cells (**Supplementary Material**). These important modifications were confirmed by TEM analysis that showed a high percentage of severely damaged SCs with nuclei deeply invaginated, shrunk, and disorder/marginalization of chromatin components, all typical features of apoptosis. In addition, we noted that endoplasmic reticulum membranes became swollen, very fragmented, or almost completely missing; mitochondrial morphology was severely affected and mitochondrial density dramatically decreased. Moreover, we observed the presence of several large vacuoles as the result of apoptotic mitochondria and/or enlarged endoplasmic reticulum.

To confirm the activation of apoptosis observed by TEM analysis, we performed the caspase-3 protein expression, crucial mediator of programmed cell death and essential for some of the characteristic changes in cell morphology associated with the execution and completion of apoptosis (40, 41). Our results, confirming previous *in vivo* (23) and *in vitro* (26) reports, showed at each concentration of NPs, a caspase-3 upregulation at the first and second week, with greater expression of p19 kDa active fragment only at toxic dose.

The increased intracellular production of ROS represents one of the most important mechanisms activated by nanomaterials. Typically, high doses of NPs, for example, 50 µg/ml in human fibroblasts (50) or 250 µg/ml in human hepatoma HepG2 (51), are associated with ROS generation.

In contrast, no intracellular production of ROS has been observed under either acute treatment (24 h), or chronic exposures (up to 4 weeks) (50) and either with the rutile and anatase forms (at a concentration of 50–500 µg/ml TiO_2_ NPs) (51), and in different cell models such as human pulmonary fibroblast (52) and Caco‐2 monolayer (at a concentration of 200 µg/ml TiO_2_ NPs) (53).

Our data would agree with these latter results. In fact, in our model, the dose of 5 µg/ml TiO_2_ NPs did not affect ROS intracellular level in contrast with the chronic exposure at the highest concentration, necessary condition to induce ROS production.

For deepening the analysis of ROS production, we evaluated the gene expression of antioxidative enzymes (ROS removal agents) including SOD, HO-1, and GHSPx as downstream molecules of Nrf2/ARE pathway (54–56). It is well known that Nrf2 is a crucial transcription factor in oxidative stress by regulating the expressions of phase II detoxification enzymes and antioxidant enzymes (56). Our results demonstrated that the exposure to TiO_2_ NPs resulted primarily in SOD1 and HO-1 upregulation at both concentrations, then, only the dose of 100 µg/ml TiO_2_ induced the upregulation of GHSPx. On this basis, we could hypothesize that the Nrf2/ARE pathway was sufficient to cope ROS production only at the subtoxic dose of TiO_2_ NPs; meanwhile, its activation was not able to counteract the oxidative stress generated at the toxic dose. As future perspective, to better understand the damage induced by ROS production, it will be useful to evaluate the involvement of the tyrosine kinase receptor HER2, involved in the epidermal growth factor-growth factor pathway (EGFG-GF), which is responsible for SCs proliferation, in addition to the assessment of ki67 and PCNA as indicators of germ cell mitotic and meiotic processes *in vivo* model (57).

Moreover, we investigated the gene expression of LDH-A as a marker of alteration of membrane integrity (26, 50), observing a significant increase only at the dose of 100 µg/ml TiO_2_ NPs, confirming the detrimental effects of exposure to toxic dose of NPs on the membrane integrity.

Oxidative DNA damage is reported as another possible effect related to ROS production. However, the supposed genotoxic potential of TiO_2_ NPs is debated because of conflicting results reported in the literature in both *in vivo* and *in vitro* models, most likely related to the different experimental conditions used.

To detect low-genotoxicity DNA damage induced by environmental contaminants, including nanomaterials, comet assay has been recognized as a reliable and sensitive technique (58, 59).

In our study, a statistically significant oxidative damage was observed at the lowest concentration of TiO_2_ NPs at 24 h, whereas, only at the highest concentration of NPs, it was observed throughout the experiment with a time-dependent relationship. On the basis of what was observed, we could speculate that in our model the increased DNA damage observed at 24 h at the lowest concentration quickly activated the DNA repair systems as already observed by Zijno et al. (60) and that chronic exposure to subtoxic doses of TiO_2_ NPs did not induce oxidative stress or genotoxic damage.

On the other hand, a significant increase of genotoxic damage before the oxidative one, observed after the exposure to toxic doses of TiO_2_ NPs, could underline as the former is a much more sensitive and early marker than the latter.

The effects of TiO_2_ NPs on SCs functionality were further evaluated by studying the levels of two specific SCs marker as AMH and inhibin B. AMH is a glycoprotein dimer belonging to the TGF-β family that is exclusively secreted by SCs, and it has become one of the most useful markers to study testicular function during the prepubertal period in males (61). Another specific and important marker of SCs action is inhibin B that provides for a negative feedback on FSH secretion and is clinically used to assess the presence and function of SCs during childhood (45).

In our study, the gene expression of both AMH and inhibin B was upregulated at 24 h and then downregulated over the course of the experiment at both concentrations, suggesting that TiO_2_ NPs treatment impaired SCs functionality as expressed by the downregulation of both protein secretions overtime, inducing, at first, a transcriptional upregulation as a physiological response followed, in the end, by a definitive breakdown. These results were in agreement with other reports on porcine prepubertal SCs treated with other heavy metals (43, 45).

Another possible effect related to nanomaterial exposure is the activation of a proinflammatory response that ultimately could induce reproductive toxicity by disturbing the immune balance and immune privilege of the testis, leading to a chronic inflammation state. Grassian et al. (62) previously reported that mice subacutely exposed to 2–5 nm TiO_2_ NPs showed a significant but moderate proinflammatory response. Park et al. (42) reported an activated inflammatory response through the upregulation of inflammatory cytokines in mouse bronchial alveolar lavage (BAL) fluid following exposure of TiO_2_ NPs. Ye et al. (27) observed an increased inflammatory response in a primary cultured rat SCs following TiO_2_ NPs administration characterized by the expression of IL-1β, TNF-α, IFN-γ, and IL-10. To evaluate the proinflammatory response, we focused on the analysis of the gene expression of IL-1α and IL-6 that showed, at 5 µg/ml of TiO_2_ NPs, an increase limited to the first, maximal second week, time necessary for SCs to put in place responses to the perturbation caused by NPs exposure, and be able to counteract them. In contrast, the dose of 100 µg/ml TiO_2_ NPs induced an obvious inflammatory state with a dose- and time-relationship increase.

Simultaneously, SCs tried to modulate an exacerbated *in vitro* proinflammatory response by the activation of the IDO-dependent mechanism TGF-β1 mediated as demonstrated by the further upregulation of TGF-β1 and IDO gene expression (63). Fallarino et al., for the first time, demonstrated the expression of IDO in SCs (63). In particular, they showed that IDO, with the involvement of the kynurenine pathway (the O_2_-dependent oxidation of L-tryptophan), plays a key role in immune modulation (64). In our *in vitro* model, we observed at the subtoxic dose that, at the third week, the immunomodulatory response tended to normalize because the proinflammatory cytokines were reduced, while at the toxic dose, was still active as a response to the proinflammatory stress.

In order to identify the intracellular signaling pathways associated with TiO_2_ NPs exposure and subsequent gene regulation, we investigated the phosphorylation ratio of some key signaling kinases by Western blot analysis. MAPKs (which are composed of three main members ERK, JNK, and p38) and PI3K/Akt signaling pathways are involved in a wide range of cellular processes, including NF-kB activation, an important nuclear transcription factor which regulates the expression of many genes involved in several cell responses such as inflammation and apoptosis (65, 66).

Treatment with both concentrations of NPs markedly increased the phosphorylation ratio of ERK1-2 up to the second week, demonstrating the involvement of this pathway in the responses initiated by the SCs after the exposure to NPs, as already reported by Han et al. in a model of porcine endothelial vascular cells treated with TiO_2_ NPs (67).

The phosphorylation ratio of JNK was upregulated only at the dose of 100 µg/ml TiO_2_ NPs from the second week of exposure throughout the end of experimentation, confirming its role as a kinase specifically activated by a significant cellular stress (66).

According to Ye et al. (27), who observed the activation of p-38 pathway in primary cultured rat SCs after an acute exposure at 5 µg/ml of TiO_2_ NPs, we demonstrated the upregulation of phosphorylation ratio of p38 at 24 h and at the lowest dose of NPs, as a response to cell proliferation.

In contrast, p38 downregulation at high dose of NPs was expression of a really stressful and toxic condition to induce apoptosis and a reduction in the number of SCs already starting from the first week as observed by TEM analysis and cell number evaluation.

Akt phosphorylation at Ser473, a well-known signal pathway with antiapoptotic function, was activated only at subtoxic concentration, throughout the whole experiment; meanwhile, the concentration of 100 µg/ml, after an early activation at 24 h, silenced its expression, confirming again its detrimental effects on SCs viability (67).

Finally, we observed the increased phosphorylation ratio of NF-kB at both concentrations starting from the second week of treatment in contrast to Ye et al. (27) who observed its upregulation already at 24 h and at concentrations ranging from 10 to 50 µg/ml.

In our model, this delayed activation could be explained by the fact that ROS production, considered a key element in the activation of this nuclear transcription factor was demonstrated only at the highest concentration of NPs from the second week. Whereas, the activation of NF-kB at subtoxic concentration during the chronic exposure could be explained by its involvement in the apoptotic death, as previously reported (68) and confirmed in our *in vitro* model subjected to long-term exposure, by TEM analysis.

## 5 Conclusions

The results of our “*in vitro*” study confirmed the negative impact of TiO_2_ NPs on porcine prepubertal SCs. In fact, SCs at a dose of 5 µg/ml seemingly maintained the morphological integrity, but a deeper analysis revealed how SCs were induced to activate a series of responses in an attempt to neutralize the harmful effects prompted by long time constant exposure that inevitably led to an altered ultrastructure, functionality, and viability.

Exposure to the dose of 100 µg/ml highlighted the negative effects of TiO_2_ NPs on the morphological-ultrastructural integrity, apoptosis, alteration of functionality, and the induction of a proinflammatory state in SCs.

Although *in vitro* studies with NPs enable the identification of conceptual models of mechanistic interaction with cells, it is very important to consider that, they do not represent a full realistic model of how NPs will interact with the specific organ of the body *in vivo*. That said however, since our model is of a superior mammal, whose physiology is very similar to that of humans, our results pave the way for studying NPs toxicology in this latter species. Moreover, our study points out the importance of deepening the effects of TiO_2_ NPs in *in vivo* animal model, especially at subtoxic dose and under chronic exposure because the impact of such a slow and insidious poisoning is not immediately perceivable and shows its side effects after a long-term treatment. Unfortunately, nowadays, no consistent epidemiologic studies exist on the association between reproductive health and the risk of TiO_2_ NPs exposure in humans. Certainly, further screenings will be necessary to verify this association. Importantly, the acquired knowledge could help to adopt containment strategies and active surveillance programs in the next future as prevention measures before irreversible damage to SCs occurs and consequently affects spermatogenesis.

## Data Availability Statement

The datasets presented in this study can be found in online repositories. The names of the repository/repositories and accession number(s) can be found below: https://doi.org/10.5061/dryad.7d7wm37wf.

## Ethics Statement

The animal studies were conducted in agreement with the guidelines adopted by the Italian Approved Animal Welfare Assurance (A-3143-01) and the European Communities Council Directive of November 24, 1986 (86/609/EEC). The experimental protocols were approved by the University of Perugia.

## Author Contributions

FM, IA, and AM designed the study and drafted the manuscript. AM synthesized TiO_2_ NPs. FM, IA, and LA performed the experimental procedures. CB performed real-time PCR and analyzed the data. CL performed WB and analyzed the data. SB and AF performed TEM. MM and DB performed ROS analysis. MM and CR performed Comet analysis. MN performed ICP-OES analysis. AG, GM, and TB gave experimental guidance. CA, SG, and GL supervised and revised the manuscript. All authors have read and agreed to the published version of the manuscript.

## Funding

This research was funded by Fondazione Cassa di Risparmio di Perugia, code of Project: 2019.0382.029.

## Conflict of Interest

The authors declare that the research was conducted in the absence of any commercial or financial relationships that could be construed as a potential conflict of interest.

## Publisher’s Note

All claims expressed in this article are solely those of the authors and do not necessarily represent those of their affiliated organizations, or those of the publisher, the editors and the reviewers. Any product that may be evaluated in this article, or claim that may be made by its manufacturer, is not guaranteed or endorsed by the publisher.

## References

[1] Powers KW Carpinone PL Siebein KN. Characterization of Nanomaterials for Toxicological Studies. Methods Mol Biol (2012) 926:13–32. doi: 10.1007/978-1-62703-002-1_2 22975954

[2] Gambelunghe A Giovagnoli S Di Michele A Boncompagni S Dell’Omo M Leopold K. Redox-Sensitive Glyoxalase 1 Up-Regulation Is Crucial for Protecting Human Lung Cells From Gold Nanoparticles Toxicity. Antioxidants (2020) 9(8):697. doi: 10.3390/antiox9080697 PMC7463694 32756399

[3] Hoet PH Brüske-Hohlfeld I Salata OV. Nanoparticles – Known and Unknown Health Risks. J Nanobiotechnol (2004) 2:12. doi: 10.1186/1477-3155-2-12 PMC544578 15588280

[4] Oberdörster G Oberdörster E Oberdörster J. Nanotoxicology: An Emerging Discipline Evolving From Studies of Ultrafine Particles. Environ Health Perspect (2005) 113(7):823–39. doi: 10.1289/ehp.7339 PMC1257642 16002369

[5] Oberdorster G. Toxicology of Air Born Environment and Occupational Particles. Part Fibre Toxicol (2006) 5(3):83–91. doi: 10.1289/ehp.7339

[6] Derfus AM Chan WCW Bhatia SN. Probing the Cytotoxicity of Semiconductor Quantum Dots. Nano Lett (2004) 4(1):11–8. doi: 10.1021/nl0347334 PMC5588688 28890669

[7] Chou CC Hsiao HY Hong QS Chen CH Peng YW Chen HW. Single-Walled Carbon Nanotubes Can Induce Pulmonary Injury in Mouse Model. Nano Lett (2008) 8(2):437–45. doi: 10.1021/nl0723634 18225938

[8] Lin P Chen JW Chang LW Wu JP Redding L Chang H. Computational and Ultrastructural Toxicology of a Nanoparticle, Quantum Dot 705, in Mice. Environ Sci Technol (2008) 42:6264–70. doi: 10.1021/es800254a 18767697

[9] Schipper ML Nakayama-Ratchford N Davis CR Kam NWS Chu P Liu Z. A Pilot Toxicology Study of Single-Walled Carbon Nanotubes in a Small Sample of Mice. Nat Nanotechnol (2008) 3:216–21. doi: 10.1038/nnano.2008.68 18654506

[10] Wu J Liu W Xue C Zhou S Lan F Bi L. Toxicity and Penetration of TiO 2 Nanoparticles in Hairless Mice and Porcine Skin After Subchronic Dermal Exposure. Toxicol Lett (2009) 191:1–8. doi: 10.1016/j.toxlet.2009.05.020 19501137

[11] Bartneck M Ritz T Keul HA Wambach M Bornemann J Gbureck U. Peptide-Functionalized Gold Nanorods Increase Liver Injury in Hepatitis. ACS Nano (2012) 6:8767–77. doi: 10.1021/nn302502u 22994679

[12] Vance ME Kuiken T Vejerano EP McGinnis SP Hochella MF Jr Rejeski D. Nanotechnology in the Real World: Redeveloping the Nanomaterial Consumer Products Inventory. Beilstein J Nanotechnol (2015) 6:1769–80. doi: 10.3762/bjnano.6.181 PMC4578396 26425429

[13] Fujishima A Zhang X Tryk DA. Ti Photocatalysis and Related Surface Phenomena. Surface Sci Rep (2008) 63:515–82. doi: 10.1016/j.surfrep.2008.10.001

[14] Wang J Chen C Liu Y Jiao F Li W Lao F. Potential Neurological Lesion After Nasal Instillation of TiO(2) Nanoparticles in the Anatase and Rutile Crystal Phases. Toxicol Lett (2008) 183(1-3):72–80. doi: 10.1016/j.toxlet.2008.10.001 18992307

[15] Trouiller B Reliene R Westbrook A Solaimani P Schiestl RH. Titanium Dioxide Nanoparticles Induce DNA Damage and Genetic Instability In Vivo in Mice. Cancer Res (2009) 69(22):8784–9. doi: 10.1158/0008-5472.CAN-09-2496 PMC3873219 19887611

[16] Elgrabli D Beaudouin R Jbilou N Floriani M Pery A Rogerieux F. Biodistribution and Clearance of TiO2 Nanoparticles in Rats After Intravenous Injection. PloS One (2015) 10(4):e0124490. doi: 10.1371/journal.pone.0124490 25909957 PMC4409301

[17] Iavicoli I Leso V Bergamaschi A. Toxicological Effects of Titanium Dioxide Nanoparticles: A Review of In Vivo Studies. J Nanomater (2012) 2012:964381. doi: 10.1155/2012/964381 21744743

[18] La Z Yang WX. Nanoparticles and Spermatogenesis: How do Nanoparticles Affect Spermatogenesis and Penetrate the Blood–Testis Barrier. Nanomed (Lond) (2012) 7:579–96. doi: 10.2217/nnm.12.20 22471721

[19] Shi HB Magaye R Castranova V Zhao JS. Titanium Dioxide Nanoparticles: A Review of Current Toxicological Data. Part Fibre Toxicol (2013) 10:15. doi: 10.1186/1743-8977-10-15 23587290 PMC3637140

[20] Guo LL Liu XH Qin DX Gao L Zhang HM Liu J,Y. Effects of Nano- Sized Titanium Dioxide on the Reproductive System of Male Mice. Zhonghua Nan Ke Xue (2009) 15:517–22 (in Chinese). 19593991

[21] Takeda K Suzuki K Ishihara A Kubo-Irie M Fujimoto R Tabata M. Nanoparticles Transferred From Pregnant Mice to Their Offspring can Damage the Genital and Cranial Nerve System. J Health Sci (2009) 55:95–102. doi: 10.1248/jhs.55.95

[22] Ema M Kobayashi N Naya M Hanai S Nakanishi J. Reproductive and Developmental Toxicity Studies of Manufactured Nanomaterials. Reprod Toxicol (2010) 30:343–52. doi: 10.1016/j.reprotox.2010.06.002 20600821

[23] Gao G Ze Y Zhao X Sang X Zheng L Ze X. Titanium Dioxide Nanoparticle-Induced Testicular Damage, Spermatogenesis Suppression, and Gene Expression Alterations in Male Mice. J Hazard Mater (2013) 258–9:133–43. doi: 10.1016/j.jhazmat.2013.04.046 23721730

[24] Jia F Sun Z Yan X Zhou B Wang J. Effect of Pubertal Nano-TiO2 Exposure on Testosterone Synthesis and Spermatogenesis in Mice. Arch Toxicol (2014) 88(3):781–8. doi: 10.1007/s00204-013-1167-5 24241477

[25] Komatsu T Tabata M Kubo-Irie M Shimizu T Suzuki K Nihei Y. The Effects of Nanoparticles on Mouse Testis Leydig Cells In Vitro. Toxicol In Vitro (2008) 22(8):1825–31. doi: 10.1016/j.tiv.2008.08.009 18805477

[26] Hong F Zhao X Si W Ze Y Wang L Zhou Y. Decreased Spermatogenesis Led to Alterations of Testis-Specific Gene Expression in Male Mice Following Nano-TiO2 Exposure. J Hazard Mater (2015) 300:718–28. doi: 10.1016/j.jhazmat.2015.08.010 26296075

[27] Ye L Hong F Ze X Li L Zhou Y Ze Y. Toxic Effects of TiO_2_ Nanoparticles in Primary Cultured Rat Sertoli Cells Are Mediated *via* a Dysregulated Ca^2+^/PKC/p38 MAPK/NF-κb Cascade. J BioMed Mater Res A (2017) 105(5):1374–82. doi: 10.1002/jbm.a.36021 28188686

[28] Wu N Hong F Zhou Y Wang Y. Exacerbation of Innate Immune Response in Mouse Primary Cultured Sertoli Cells Caused by Nanoparticulate TiO_2_ Involves the TAM/TLR3 Signal Pathway. J BioMed Mater Res A (2017) 105(1):198–208. doi: 10.1002/jbm.a.35906 27643721

[29] Vales G Rubio L Marcos R. Long-Term Exposures to Low Doses of Titanium Dioxide Nanoparticles Induce Cell Transformation, But Not Genotoxic Damage in BEAS-2B Cells. Nanotoxicology (2015) 9(5):568–78. doi: 10.3109/17435390.2014.957252 25238462

[30] Ammar I Wanichaya M Wisanu P. Anatase/Rutile TiO2 Composite Prepared *via* Sonochemical Process and Their Photocatalytic Activity. Mater Today: Proc (2017) 4(5):6159–65. doi: 10.1016/j.matpr.2017.06.110

[31] Zhang Y Chen Y Westerhoff P Hristovski K Crittenden JC. Stability of Commercial Metal Oxide Nanoparticles in Water. Water Res (2008) 42(8-9):2204–12. doi: 10.1016/j.watres.2007.11.036 18164742

[32] De Monte V Staffieri F Di Meo A Vannucci J Bufalari A. Comparison of Ketamine-Dexmedetomidine-Methadone and Tiletamine-Zolazepam-Methadone Combinations for Short-Term Anaesthesia in Domestic Pig. Vet J (2015) 205(3):364–8. doi: 10.1016/j.tvjl.2015.05.011 26070949

[33] Luca G Mancuso F Calvitti M Arato I Falabella G Bufalari A. Long-Term Stability, Functional Competence, and Safety of Microencapsulated Specific Pathogen-Free Neonatal Porcine Sertoli Cells: A Potential Product for Cell Transplant Therapy. Xenotransplantation (2015) 22(4):273–83. doi: 10.1111/xen.12175 26134468

[34] Arato I Luca G Mancuso F Bellucci C Lilli C Calvitti M. An “*In Vitro*” Prototype of a Porcine Biomimetic Testis-Like Cell Culture System: A Novel Tool for the Study of Reassembled Sertoli and Leydig Cells. Asian J Androl (2018) 20(2):160–5. doi: 10.4103/aja.ja_47_17 PMC5858101 29148520

[35] Tice RR Agurell E Anderson D Burlinson B Hartmann A Kobayashi H. Single Cell Gel/COMET Assay: Guidelines for *In Vitro* and *In Vivo* Genetic Toxicology Testing, Environ. Mol Mutagen (2000) 35(3):206–21. doi: 10.1002/(SICI)1098-2280(2000)35:3b206::AIDEM8N3.0.CO;2-J 10737956

[36] Giovagnoli S Mancuso F Vannini S Calvitti M Piroddi M Pietrella D. ‘Microparticle-Loaded Neonatal Porcine Sertoli Cells for Cell-Based Therapeutic and Drug Delivery System’. J Control Release (2014) 192:249–61. doi: 10.1016/j.jconrel.2014.08.001 25111130

[37] Arato I Milardi D Giovagnoli S Grande G Bellucci C Lilli C. “*In "Vitro"* Lps-Stimulated Sertoli Cells Pre-Loaded With Microparticles: Intracellular Activation Pathways. Front Endocrinol (2021) 11:611932. doi: 10.3389/fendo.2020.611932 PMC7817811 33488524

[38] Antognelli C Mancuso F Frosini R Arato I Calvitti M Calafiore R. Testosterone and Follicle Stimulating Hormone-Dependent Glyoxalase 1 Up-Regulation Sustains the Viability of Porcine Sertoli Cells Through the Control of Hydroimidazolone- and Argpyrimidine-Mediated NF-κb Pathway. Am J Pathol (2018) 188(11):2553–63. doi: 10.1016/j.ajpath.2018.07.01 30125541

[39] Bradford MM. A Rapid and Sensitive Method for the Quantitation of Microgram Quantities of Protein Utilizing the Principle of Protein-Dye Binding. Anal Biochem (1976) 72:248–54. doi: 10.1016/0003-2697(76)90527-3 942051

[40] Fettucciari K Ponsini P Gioè D Macchioni L Palumbo C Antonelli E. Enteric Glial Cells Are Susceptible to *Clostridium Difficile* Toxin B. Cell Mol Life Sci (2017) 74:1527–155. doi: 10.1007/s00018-016-2426-4 PMC11107567 27891552

[41] Valencia-Cruz G Shabala L Delgado-Enciso I Shabala S Bonales-Alatorre E Pottosin II. K Bg and Kv1.3 Channels Mediate Potassium Efflux in the Early Phase of Apoptosis in Jurkat T Lymphocytes. Am J Physiol Cell Physiol (2009) 297:C1544–53. doi: 10.1152/ajpcell.00064.2009 19794143

[42] Park EJ Yoon J Choi K Yi J Park K. Induction of Chronic Inflammation in Mice Treated With Titanium Dioxide Nanoparticles by Intratracheal Instillation. Toxicology (2009) 260(1-3):37–46. doi: 10.1016/j.tox.2009.03.005 19464567

[43] Mancuso F Arato I Lilli C Bellucci C Bodo M Calvitti M. Acute Effects of Lead on Porcine Neonatal Sertoli Cells. Vitro Toxicol In Vitro (2018) 48:45–52. doi: 10.1016/j.tiv.2017.12.013 29273543

[44] Marinucci L Balloni S Bellucci C Lilli C Stabile AM Calvitti M. Effects of Nicotine on Porcine Pre-Pupertal Sertoli Cells: An *In Vitro* Study. Toxicol In Vitro (2020) :67:104882. doi: 10.1016/j.tiv.2020.104882 32423882

[45] Luca G Lilli C Bellucci C Mancuso F Calvitti M Arato I. Toxicity of Cadmium on Sertoli Cell Functional Competence: An In Vitro Study. J Biol Regul Homeost Agents (2013) 27(3):805–16. 24152845

[46] Grande F Tucci P. Titanium Dioxide Nanoparticles: A Risk for Human Health? Mini Rev Med Chem (2016) 16(9):762–9. doi: 10.2174/1389557516666160321114341 26996620

[47] Liang G Pu Y Yin L Liu R Ye B Su Y. Influence of Different Sizes of Titanium Dioxide Nanoparticles on Hepatic and Renal Functions in Rats With Correlation to Oxidative Stress. J Toxicol Environ Health A (2009) 72(11-12):740–5. doi: 10.1080/15287390902841516 19492237

[48] De Jong WH Borm PJ. Drug Delivery and Nanoparticles: Applications and Hazards. Int J Nanomed (2008) 3(2):133–49. doi: 10.2147/ijn.s596 PMC2527668 18686775

[49] Takahashi Y Mizuo K Shinkai Y Oshio S Takeda K. Prenatal Exposure to Titanium Dioxide Nanoparticles Increases Dopamine Levels in the Prefrontal Cortex and Neostriatum of Mice. J Toxicol Sci (2010) 35(5):749–56. doi: 10.2131/jts.35.749 20930469

[50] Huang S Chueh PJ Lin YW Shih TS Chuang SM. Disturbed Mitotic Progression and Genome Segregation Are Involved in Cell Transformation Mediated by Nano-TiO2 Long-Term Exposure. Toxicol Appl Pharmacol (2009) 241(2):182–94. doi: 10.1016/j.taap.2009.08.013 19695278

[51] Petković J Küzma T Rade K Novak S Filipič M. Pre-Irradiation of Anatase TiO2 Particles With UV Enhances Their Cytotoxic and Genotoxic Potential in Human Hepatoma HepG2 Cells. J Hazard Mater (2011) 196:145–52. doi: 10.1016/j.jhazmat.2011.09.004 21945684

[52] Armand L Dagouassat M Belade E Simon-Deckers A Le Gouvello S Tharabat C. Titanium Dioxide Nanoparticles Induce Matrix Metalloprotease 1 in Human Pulmonary Fibroblasts Partly via an Interleukin-1β-Dependent Mechanism. Am J Respir Cell Mol Biol (2013) 48(3):354–63. doi: 10.1165/rcmb.2012-0099OC 23239492

[53] Gerloff K Fenoglio I Carella E Kolling J Albrecht C Boots AW. Distinctive Toxicity of TiO2 Rutile/Anatase Mixed Phase Nanoparticles on Caco-2 Cells. Chem Res Toxicol (2012) 3):646–55. doi: 10.1021/tx200334k 22263745

[54] Gonzales S Perez MJ Perazzo JC Tomaro ML. Antioxidant Role of Heme Oxygenase-1 in Prehepatic Portal Hypertensive Rats. World J Gastroenterol (2006) 12(26):4149–55. doi: 10.3748/wjg.v12.i26.4149 PMC4087362 16830363

[55] Maines MD Kappas A. Studies on the Mechanism of Induction of Haem Oxygenase by Cobalt and Other Metal Ions. Biochem J (1976) 154(1):125–31. doi: 10.1042/bj1540125 PMC1172683 819007

[56] Yang SH Yu LH Li L Guo Y Zhang Y Long M. Protective Mechanism of Sulforaphane on Cadmium-Induced Sertoli Cell Injury in Mice Testis *via* Nrf2/ARE Signaling Pathway. Molecules (2018) 23:1774. doi: 10.3390/molecules23071774 PMC6100605 30029485

[57] Ajayi AF Akhigbe RE. *In Vivo* Exposure to Codeine Induces Reproductive Toxicity: Role of HER2 Andp53/Bcl-2 Signaling Pathway. Heliyon (2020) 6:e05589. doi: 10.1016/j.heliyon.2020.e05589 33294712 PMC7695972

[58] Dusinska M Collins AR. The Comet Assay in Human Biomonitoring: Gene-Environment Interactions. Mutagenesis (2008) 23(3):191–205. doi: 10.1093/mutage/gen007 18326867

[59] Magdolenova Z Lorenzo Y Collins A Dusinska M. Can Standard Genotoxicity Tests be Applied to Nanoparticles? J Toxicol Environ Health A (2012) 75(13-15):800–6. doi: 10.1080/15287394.2012.690326 22788367

[60] Zijno A De Angelis I De Berardis B Andreoli C Russo MT Pietraforte D. Different Mechanisms Are Involved in Oxidative DNA Damage and Genotoxicity Induction by ZnO and TiO2 Nanoparticles in Human Colon Carcinoma Cells. Toxicol In Vitro (2015) 29(7):1503–12. doi: 10.1016/j.tiv.2015.06.009 26079941

[61] Josso N Rey RA Picard JY. Anti-Müllerian Hormone: A Valuable Addition to the Toolbox of the Pediatric Endocrinologist. Int J Endocrinol (2013) 2013:674105. doi: 10.1155/2013/674105 24382961 PMC3870610

[62] Grassian VH O’shaughnessy PT Adamcakova-Dodd A Pettibone J--Thorne PS. Inhalation Exposure Study of Titanium Dioxide Nanoparticles With a Primary Particle Size of 2 to 5 Nm. Environ Health Perspect (2007) 115(3):397–402. doi: 10.1289/ehp.9469 17431489 PMC1849915

[63] Fallarino F Luca G Calvitti M Mancuso F Nastruzzi C Fioretti MC. Therapy of Experimental Type 1 Diabetes by Isolated Sertoli Cell Xenografts Alone. J Exp Med (2009) 206:2511–26. doi: 10.1084/jem.20090134 PMC2768846 19822646

[64] Mellor AL Munn DH. IDO Expression by Dendritic Cells: Tolerance and Tryptophan Catabolism. Nat Rev Immunol (2004) 4:762–74. doi: 10.1038/nri1457 15459668

[65] Sun Y Liu WZ Liu T Feng X Yang N Zhou HF. Signaling Pathway of MAPK/ERK in Cell Proliferation, Differentiation, Migration, Senescence and Apoptosis. J Recept Signal Transduct Res (2015) 35(6):600–4. doi: 10.3109/10799893.2015.1030412 26096166

[66] Zhang W Liu HT. MAPK Signal Pathways in the Regulation of Cell Proliferation in Mammalian Cells. Cell Res (2002) 12(1):9–18. doi: 10.1038/sj.cr.7290105 11942415

[67] Han SG Newsome B Hennig B. Titanium Dioxide Nanoparticles Increase Inflammatory 750 Responses in Vascular Endothelial Cells. Toxicology (2013) 306:1–8. doi: 10.1016/j.tox.2013.01.014 23380242 PMC3631470

[68] Liu X Sun J. Endothelial Cells Dysfunction Induced by Silica Nanoparticles Through Oxidative Stress *via* JNK/P53 and NF-kappaB Pathways. Biomaterials (2010) 31:8198–209. doi: 10.1016/j.biomaterials.2010.07.069 20727582

