# Supplementary figures and images for: Effects of Titanium Dioxide Nanoparticles on Porcine Prepubertal Sertoli Cells: An “In Vitro” Study
Source: Front Endocrinol (Lausanne). 2022 Jan 3;12:751915. doi: 10.3389/fendo.2021.751915 (PMC8762334; doi:10.3389/fendo.2021.751915)

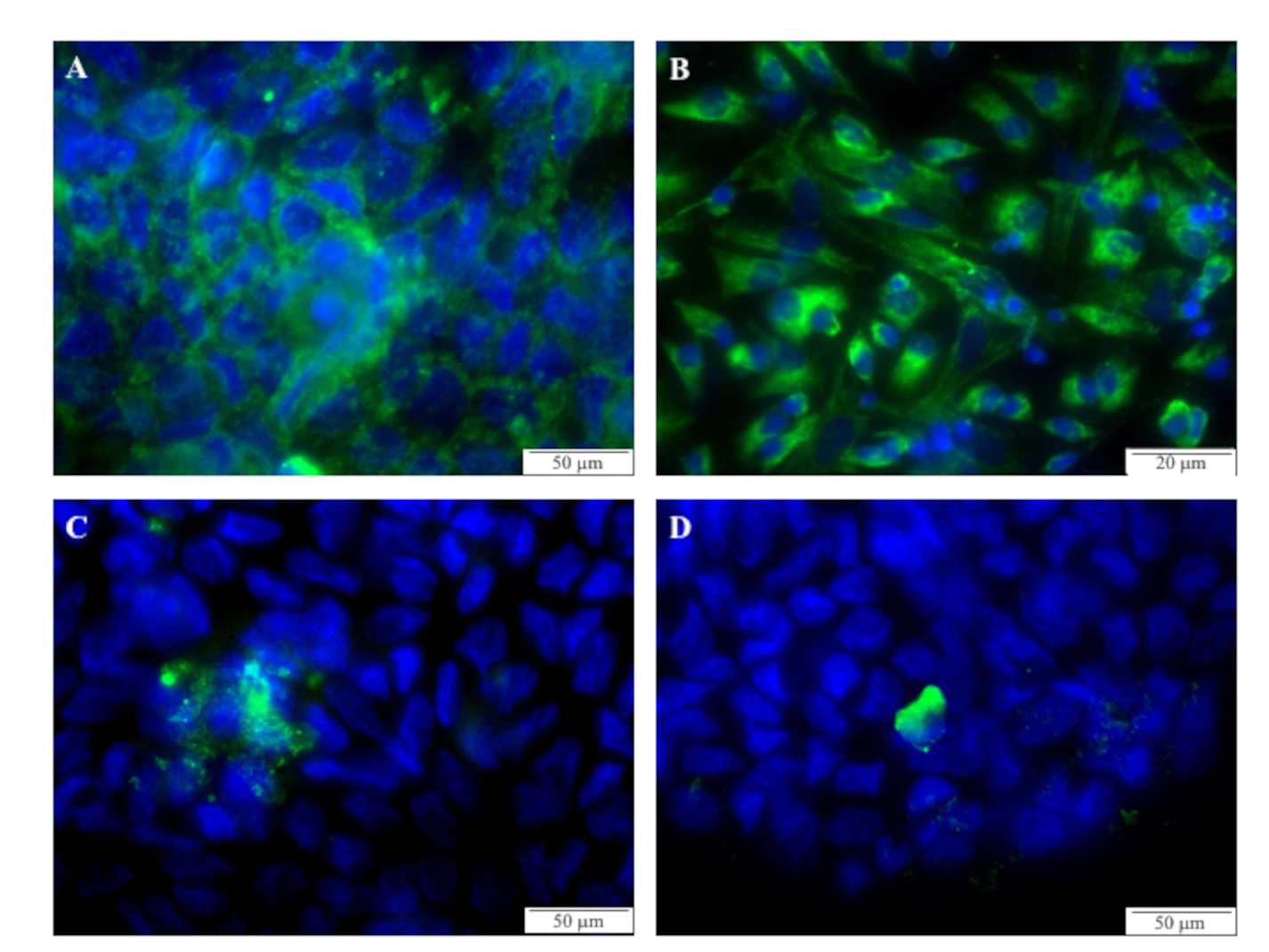

Supplement: Supplementary file 1 [file Image_1.tif]

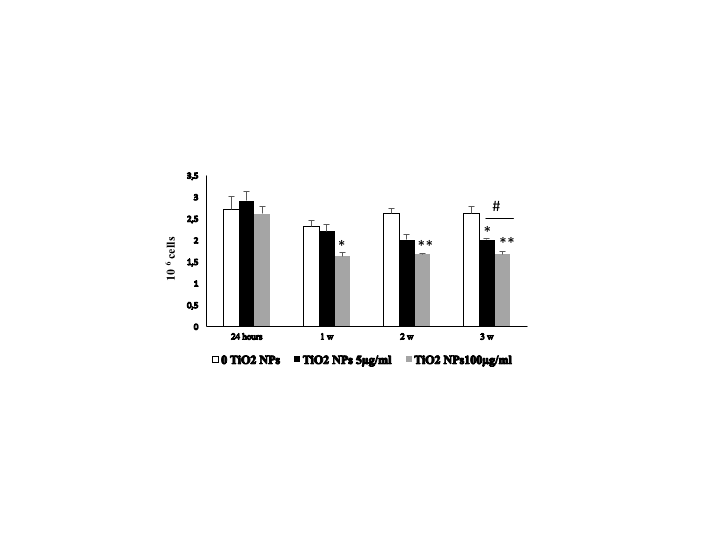

Supplement: Supplementary file 2 [file Image_2.tif]

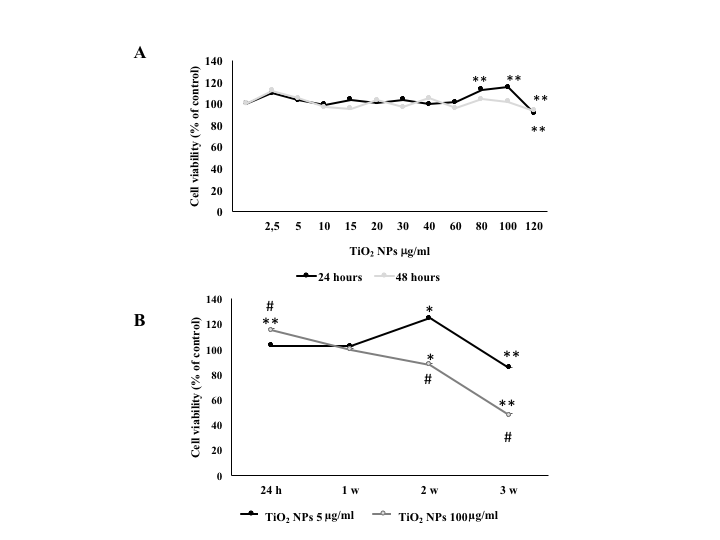

Supplement: Supplementary file 3 [file Image_3.tif]
