# Supplementary material for: Effects of Titanium Dioxide Nanoparticles on Porcine Prepubertal Sertoli Cells: An “In Vitro” Study
Source: Front Endocrinol (Lausanne). 2022 Jan 3;12:751915. doi: 10.3389/fendo.2021.751915 (PMC8762334; doi:10.3389/fendo.2021.751915)
Supplement: Supplementary file 4 [file DataSheet_1.docx]

Supplementary Material

*1 NPs suspension*

The dispersion in the stock solution used for the experimental protocol was achieved by increasing the sonication time to 30 minutes; further increase in sonication time did not produce a significant increase in dispersity (Supplementary Table 1).

For cell cultures, DMEM was found to be better than HAM-F12 (Supplementary Table 2) for dispersing TiO_2_ NPs, as reported in the literature [1]. This is due to the higher concentration of phosphate ions present in HAM F12, which thank to their strong negative charge, apply a repulsive force on TiO_2_ particles, weakly negatively charged, causing a change in the Stern layer, partially canceling the Z potential and causing the aggregation of particles [2].

The dispersion in the culture medium can be facilitated by adding a dispersing agent such as BSA, which in the presence of bivalent cations (Ca^+2^, Mg^+2^) is able to adsorb on the surface of the particles favoring their dispersion [2].

The optimal concentration of BSA for the suspension resulted 2 mg/ml, which enabled the production of a stable dispersion with particles having an average diameter of 392 nm, in line with the data found in the literature [3].

Therefore, DMEM (Euroclone, Milan, Italy) supplemented with 0.166 nM retinoic acid (Sigma–Aldrich Co., St. Louis, MO, USA), 5 mL/500 ml of insulin, ITS (cat. no. 354352, BD Biosciences, [Franklin Lakes, New Jersey](https://en.wikipedia.org/wiki/Franklin_Lakes,_New_Jersey), USA), and 2 mg/ml BSA (Sigma–Aldrich Co., St. Louis, MO, USA) was chosen as the optimal medium to perform the experimental protocol (colture medium), (Supplementary Table 2).

*2 SCs isolation, culture and characterization*

SCs were isolated according to previously established methods and substantially modified in our laboratory [33,34 main text]. Briefly, testis pairs from 7 to 15 days old anesthetized Danish Duroc piglets, after removal of the fibrous capsule, were finely chopped and enzymatically digested twice with a mixed solution of trypsin and DNase I (Sigma–Aldrich Co., St. Louis, MO, USA), and Collagenase P (Roche Diagnostics S.p.A., Monza, Italy). After twice washing in HBSS (Sigma–Aldrich Co., St. Louis, MO, USA), the tissue pellet was centrifuged and passed through a 500 mm stainless steel mesh and resuspended in glycine buffer (Sigma-Aldrich Co., St. Louis, MO, USA) [33 main text], to eliminate residual Leydig and peritubular cells. The partially digested seminiferous tubules, associated with disappearance of the peritubular cells, were collected and maintained in HAM-F12 (Euroclone, Milan, Italy) culture medium supplemented with 0.166 nM retinoic acid (Sigma–Aldrich Co., St. Louis, MO, USA), and 5 mL/500 ml of insuline transferrine selenium (ITS; Becton Dickinson cat. no. 354352) in 95% air 5% CO2 at 37°C. Following 3 days culture, SCs were incubated with 10 mM tris(hydroxymethyl)aminomethane hydrochloride (TRIS) buffer (Sigma–Aldrich Co., St. Louis, MO, USA), as previously described, to eliminate residual germinal cells [33,34 main text]. To detect the presence of AMH and vimentin (SCs marker), 3β-Hydroxysteroid dehydrogenase (3βHSD) (Leydig cells marker), and alpha smooth muscle actin (ASMA) (peritubular cells marker), immunostainings were performed according to previously reported methods [33,34 main text]. Briefly, unexposed and exposed SCs monolayers were grown on glass chamber slides (LabTek II, Nunc, Thermo Fisher, Rochester, NY, USA), and fixed in ice-cold methanol for 15 min. The fixed cells were then permeabilised (PBS, 0.2% Triton X-100), (Sigma–Aldrich Co., St. Louis, MO, USA), for 10 min at room temperature, and blocked with 0.5% BSA (Sigma-Aldrich Co., St. Louis, MO, USA) in PBS for 1 h prior to exposure to the primary antibody at +4 °C overnight. The cells were then washed in PBS three times (5 min each time) and then exposed to a secondary Alexa 488-conjugated donkey anti-goat antibody (Molecular Probes, NY, USA, 1:500) and Alexa 488-conjugated donkey anti-rabbit antibody (Molecular Probes, NY, USA, 1:500). The cells were then treated with RNAse (10 mg/ml, Sigma-Aldrich Co., St. Louis, MO, USA) and counterstained for 1 min with DAPI (Sigma-Aldrich Co., St. Louis, MO, USA). Negative controls were included without the primary antibody treatment. The cells were mounted with ProLong® Gold antifade reagent (Molecular Probes, NY, USA). To evaluate the percentage of AMH, vimentin, 3βHSD, and ASMA positive cells, chamber slides were analyzed using a BX-41 microscope (Olympus, Tokyo, Japan) equipped with a fluorescence photocamera. The isolated SCs culture was 95% pure as indicated by immunostaining for AMH and vimentin (Fig. 1S, panel A-B, respectively) with an extremely low percentage of non-SC cells (< 5%) characterised by immunostaining for 3β-HSD (Leydig cells, supplementary Fig. 1 panel C) and ASMA (peritubular cells, Figure 1S, panel D).

*3 Cell number*

The number of unexposed and exposed SCs was evaluated by the trypan blue exclusion assay. Detached cells were pelleted by centrifugation at 800 g for 5 min and mixed with 0.4% trypan blue (Sigma-Aldrich Co., St. Louis, MO, USA) in a 1:1 ratio; the cells were then counted using an automated cell counter (Invitrogen, Carlsbad, CA, USA). The dose of 5 μg/ml of NPs induced a statistically significant decrease of cell number respect to the unexposed SCs only at the third week, meanwhile, the dose of 100 μg/ml induced a significant decrease starting with the first week (Figure 2S, *p < 0.05, **p<0.001). Finally, at the third week, the dose of 100 μg/ml of NPs exhibited a significant decrease of cell number respect to the dose of 5 μg/ml, evident expression of higher toxicity (Fig. 2S, #p < 0.05).

*4 TiO_2_ NPs citotossicity*

TiO_2_ NPs citotossicity was evaluated by the 3-(4, 5-Dimethyl-thiazol-2-yl)22,5-diphenyl-tetrazolium bromide (MTT) (Sigma-Aldrich Co., St. Louis, MO, USA) test on unexposed and exposed SCs. TiO_2_ NPs at the concentrations of 2.5, 5, 15, 30, 45, 60 and 120 μg/ml were added to each well and cultured for additional 24 or 48 h. After that 10 μl MTT solution (5 mg/ml) was added to each well and further incubated for 4 h. The supernatants were removed before adding 100 μl dimethyl sulfoxide (DMSO) (Sigma-Aldrich Co., St. Louis, MO, USA) to dissolve the formazan crystal at 37°C for 30 minutes. The absorbance was measured on an automated microplate reader (Sunrise, Tecan, Männedorf, Switzerland) at 570 nm. Unexposed (0 TiO_2_ NPs μg/ml) SCs served as controls. Viability was expressed as a percentage with respect to unexposed SCs (NPs-exposed SCs ×100/ unexposed SCs). The sub-toxic dose of 5 μg/ml and the toxic dose of 100 μg/ml were chosen for all subsequent experiments at 24 hours (acute exposure) and 1, 2, 3 weeks (chronic exposure) and MTT assay was performed at each experimental time-point. For the preliminary study, at 24 hours, as shown in Figure 3S, panel A, TiO_2_ NPs did not significantly affect cell viability until 100 μg/ml, in fact, as observed, cell viability remained higher than 80% (Figure 3S, panel A), suggesting that TiO2 NPs have minimal cytotoxicity. At the doses of 80 and 100 μg/ml, an increase of viable cells was observed (Figure 3S panel A, **p<0.001 *vs* unexposed SCs), which might suggest an adaptive response by the cell with increasing concentration of TiO_2_ NPs. The percentage of metabolically active cells was statistically significantly reduced at the highest dose of 120 μg/ml, which therefore appeared to be toxic (Supplementary Figure 3 panel A, **p<0.001 *vs* unexposed SCs).

At 48 hours, the cells did not show substantial differences compared to the unexposed SCs, confirming however the toxic effect of the TiO_2_ NPs at a concentration of 120 μg/ml (Supplementary Figure 3 panel A, **p<0.001 *vs* unexposed SCs). These data would seem to indicate that after the initial stress at 24 hours, and at the highest concentrations, SCs had perfectly recovered their metabolic activity at the 48 hours, however, demonstrating a clear damage at the highest dose of 120 μg/ml. According to those results, we selected the sub toxic dose of 5 μg/ ml and the toxic dose of 100 μg/ ml to perform the experimental protocol with an acute (24 hours) and chronic exposure (from 1 up to 3 week) time (Figure 3S panel A).

MTT assays performed during the 3 weeks of treatment with TiO_2_ NPs showed an increase in metabolically active cells at the dosage of 5 μg/ml at the second week (Figure 3S panel B *p<0.05 *vs* unexposed SCs). This effect could be due to a defensive mechanism put in place by the cell in response to a noxious stimulus, which however does not seem to be sufficient to preserve the cell from the statistically significant reduction in the percentage of metabolically active cells of 20% observed at the third week compared to the unexposed SCs), (Figure 3S panel B, **p<0.001 *vs* unexposed SCs).

The concentration of 100 μg/ml showed an increase in the percentage of metabolically active cells at 24 hours, again as an early response to NPs toxicity as previously observed at the lowest dose, and clear signs of toxicity from the second week of exposure (*p<0.05 *vs* unexposed SCs; # *vs* 5 μg/ml of TiO_2_ NPs). At the third week, the number of metabolically active cells was reduced more than 50% compared to the unexposed SCs and 30% respect to 5 μg/ml of TiO_2_ NPs clear expression of increased toxicity as the concentration increased), (Figure 3S panel B, **p<0.001 *vs* unexposed SCs; # *vs* 5 μg/ml of TiO_2_ NPs).

**Figure 1S. Characterization of “*in vitro”* cultured pre-pubertal porcine SCs monolayers by fluorescence microscopy.** (A) SCs after immunostaining with AMH antibody and visualized by anti-goat Alexa Fluor 488 (green). (B) SCs after immunostaining with vimentin antibody and visualized by anti-goat Alexa Fluor 488 (green). (C) SCs after immunostaining with 3βHSD antibody and visualized by anti-rabbit Alexa Fluor 488 (green). (D) SCs, after immunostaining with ASMA antibody and visualized by anti-rabbit Alexa Fluor 488 (green). Nuclei are counterstained with 4′,6-diamidino-2-phenylindole (DAPI) (blue). The images are representative of three independent experiments.

**Figure 2S.** **Cells number.** (A) Cells number evaluated by trypan blue exclusion assay in unexposed SCs (white bar) and exposed to TiO_2_ NPs 5 (black bar) and 100 μg/ml (grey bar) for 24 hours, 1, 2 and 3 weeks. Data represented as mean ± S.E.M. (*p<0.05 and **p<0.001 *vs* unexposed SCs; #p < 0.05 *vs* 5 μg/ml of TiO_2_ NPs of three independent experiments, each performed in triplicate).

**Figure 3S.** **Evaluation of TiO2 NPs tossicity by MTT test.** (A) Evaluation of TiO_2_ NPs tossicity in SCs at 24 (black line) and 48 hours (grey line) of incubation with TiO_2_ NPs 0, 2.5, 5, 15, 30, 45, 60, or 120 μg/ml in a preliminary experiment performed to assess the range of sub-toxic and toxic doses to be used in the experiment. (B) Evaluation of TiO_2_ NPs tossicity in SCs at 24 hours, 1, 2 and 3 weeks of incubation with TiO_2_ NPs 5 (black line) and 100 μg/ml (grey line). Data represented as mean ± S.E.M. (*p<0.05 and **p<0.001 *vs* unexposed SCs; #p < 0.05 *vs* 5 μg/ml of TiO_2_ NPs of three independent experiments, each performed in triplicate).

| Sonication time | Mean diameter | Index of polydispersity |
| --- | --- | --- |
| 15 min | 270 nm | 0.091 |
| 30 min | 270 nm | 0.294 |

**Supplementary Table 1**. Mean diameter and index of polydispersity of TiO_2_ NPs in endotoxin free water

| Medium | Mean diameter | Index of polydispersity |
| --- | --- | --- |
| HAM-F12 | 1661 nm | 0.6 |
| DMEM | 492 nm | 0.406 |
| Colture medium | 392 nm | 0.2 |

**Supplementary Table 2.** Mean diameter and index of polydispersity of TiO_2_ NPs at a concentration of 10 μg/ml in colture medium

**References**

- - - 1. Zhang, Y., Chen, Y., Westerhoff , P., Hristovski K., Crittenden, J.C. Stability of commercial metal oxide nanoparticles in water. *Water Res.* 2008 Apr;42(8-9):2204-12. doi: 10.1016/j.watres.2007.11.036.
      2. Hunter, R. J. Zeta Potential in Colloid Science 1st edition. Editors: R. H. Ottewill R. L. Rowell. eBook ISBN: 9781483214085, Paperback ISBN: 9780123619617, Academic Press, 9th December 1988.
      3. Ji, Z.,  [Jin](https://pubs.acs.org/action/doSearch?field1=Contrib&text1=Xue++Jin), X.,  [George](https://pubs.acs.org/action/doSearch?field1=Contrib&text1=Saji++George), S., [Xia](https://pubs.acs.org/action/doSearch?field1=Contrib&text1=Tian++Xia), T.,  [Meng](https://pubs.acs.org/action/doSearch?field1=Contrib&text1=Huan++Meng), H.,  [Wang](https://pubs.acs.org/action/doSearch?field1=Contrib&text1=Xiang++Wang), X.,  [Suarez](https://pubs.acs.org/action/doSearch?field1=Contrib&text1=Elizabeth++Suarez), E.,  [Zhang](https://pubs.acs.org/action/doSearch?field1=Contrib&text1=Haiyuan++Zhang), H.,  [Hoek](https://pubs.acs.org/action/doSearch?field1=Contrib&text1=Eric+M.V.++Hoek), E.M.V., [Godwin](https://pubs.acs.org/action/doSearch?field1=Contrib&text1=Hilary++Godwin), H.,  [Nel](https://pubs.acs.org/action/doSearch?field1=Contrib&text1=Andr%C3%A9+E.++Nel), A.E., [Zink](https://pubs.acs.org/action/doSearch?field1=Contrib&text1=Jeffrey+I.++Zink), J.I. . Dispersion and Stability Optimization of TiO2 Nanoparticles in Cell Culture Media, Published in final edited form as: *Environ Sci Technol* 201044(19): 7309–7314. Httpp://doi:10.1021/es100417s.
